# Long Non-Coding RNA- Associated Competing Endogenous RNA Axes in T-Cells in Multiple Sclerosis

**DOI:** 10.3389/fimmu.2021.770679

**Published:** 2021-12-08

**Authors:** Hani Sabaie, Zoha Salkhordeh, Mohammad Reza Asadi, Soudeh Ghafouri-Fard, Nazanin Amirinejad, Mahla Askarinejad Behzadi, Bashdar Mahmud Hussen, Mohammad Taheri, Maryam Rezazadeh

**Affiliations:** ^1^ Molecular Medicine Research Center, Tabriz University of Medical Sciences, Tabriz, Iran; ^2^ Department of Medical Genetics, Faculty of Medicine, Tabriz University of Medical Sciences, Tabriz, Iran; ^3^ Department of Medical Genetics, Faculty of Medicine, Kerman University of Medical Sciences, Kerman, Iran; ^4^ Student Research Committee, Tabriz University of Medical Sciences, Tabriz, Iran; ^5^ Department of Medical Genetics, School of Medicine, Shahid Beheshti University of Medical Sciences, Tehran, Iran; ^6^ Department of Biology, Faculty of Sciences, Shahid Bahonar University of Kerman, Kerman, Iran; ^7^ Department of Pharmacognosy, College of Pharmacy, Hawler Medical University, Erbil, Iraq; ^8^ Men’s Health and Reproductive Health Research Center, Shahid Beheshti University of Medical Sciences, Tehran, Iran; ^9^ Institute of Human Genetics, Jena University Hospital, Jena, Germany

**Keywords:** bioinformatics analysis, competing endogenous RNA, long non-coding RNA, microarray, multiple sclerosis

## Abstract

Multiple sclerosis (MS) is an immune-mediated demyelinating and degenerative disease with unknown etiology. Inappropriate response of T-cells to myelin antigens has an essential role in the pathophysiology of MS. The clinical and pathophysiological complications of MS necessitate identification of potential molecular targets to understand the pathogenic events of MS. Since the functions and regulatory mechanisms of long non-coding RNAs (lncRNAs) acting as competing endogenous RNAs (ceRNAs) in MS are yet uncertain, we conducted a bioinformatics analysis to explain the lncRNA-associated ceRNA axes to clarify molecular regulatory mechanisms involved in T-cells responses in MS. Two microarray datasets of peripheral blood T-cell from subjects with relapsing-remitting MS and matched controls containing data about miRNAs (GSE43590), mRNAs and lncRNAs (GSE43591) were downloaded from the Gene Expression Omnibus database. Differentially expressed miRNAs (DEmiRNAs), mRNAs (DEmRNAs), and lncRNAs (DElncRNAs) were identified by the limma package of the R software. Protein-protein interaction (PPI) network and module were developed using the Search Tool for the Retrieval of Interacting Genes/Proteins (STRING) and the Molecular Complex Detection (MCODE) Cytoscape plugin, respectively. Using DIANA-LncBase and miRTarBase, the lncRNA-associated ceRNA axes was constructed. We conducted a Pearson correlation analysis and selected the positive correlations among the lncRNAs and mRNAs in the ceRNA axes. Lastly, DEmRNAs pathway enrichment was conducted by the Enrichr tool. A ceRNA regulatory relationship among Small nucleolar RNA host gene 1 (*SNHG1*), *hsa-miR-197-3p*, YOD1 deubiquitinase (*YOD1*) and zinc finger protein 101 (*ZNF101*) and downstream connected genes was identified. Pathway enrichment analysis showed that DEmRNAs were enriched in “Protein processing in endoplasmic reticulum” and “Herpes simplex virus 1 infection” pathways. To our knowledge, this would be the first report of a possible role of *SNHG1*/*hsa-miR-197-3p*/*YOD1*/*ZNF101* axes in the pathogenesis of MS. This research remarks on the significance of ceRNAs and prepares new perceptions for discovering the molecular mechanism of MS.

## Introduction

Multiple sclerosis (MS) is the most frequent cause of non-traumatic neurological disability in young adult people (1). Estimates indicate that a total of 2.8 million individuals with MS live around the world (35.9 per 100,000 population) (2). In this degenerative disorder, the central nervous system is demyelinated through the mediation of the immune system. Yet, the main cause of MS is not known. MS presents in four clinical types: relapsing-remitting MS (RRMS), secondary progressive MS (SPMS), primary progressive MS (PPMS), and progressive relapsing MS (PRMS). RRMS is the most common subtype, which is represented by acute attacks (relapses) and then partially or fully recovered phases (remission) (3). Although the causes and etiologies underlie MS are not completely apprehended, there are proofs that it originates from multiple factors involving central and peripheral immunological tolerance mechanisms. An inappropriate T-cell response to myelin antigens is probably an important mechanism in MS pathogenesis (4). Emerging evidence indicates the potential of non-coding RNAs, especially long non-coding RNAs (lncRNAs) and the microRNAs (miRNAs), in the regulation of gene expression, providing novel prospects to understand the progression of MS (5–7). LncRNAs have major contributions to complex disorders (e.g., MS) through functioning as competing endogenous RNAs (ceRNAs) (8).

The ceRNA hypothesis suggests a cross-talk between both coding and non-coding RNAs through miRNA response elements (MREs), as miRNA complementary sequences, thereby forming a large-scale regulatory network in various parts of the transcriptome. Based on this supposition, through ceRNA regulatory mechanism, these two RNA transcripts will be indirectly correlated with miRNAs levels. Additionally, expression levels of these two RNA transcripts are positively correlated with each other (9). The consequence of disrupted balance of ceRNA cross-talk is well documented in a variety of disorders (10). Nevertheless, the roles and regulatory mechanisms of lncRNAs acting as ceRNAs in MS are still unclear.

As MS is complex in terms of both pathophysiological and clinical aspects, it is necessary to identify wide-ranging potential molecular targets to understand the pathogenic processes involved in MS. As the functions and regulatory mechanisms of lncRNAs role as ceRNAs in MS is not clear, bioinformatics analysis was done to clarify the lncRNA-associated ceRNA axes to explain molecular regulatory mechanisms involved in T-cells in MS.

## Methods

**Figure 1:**
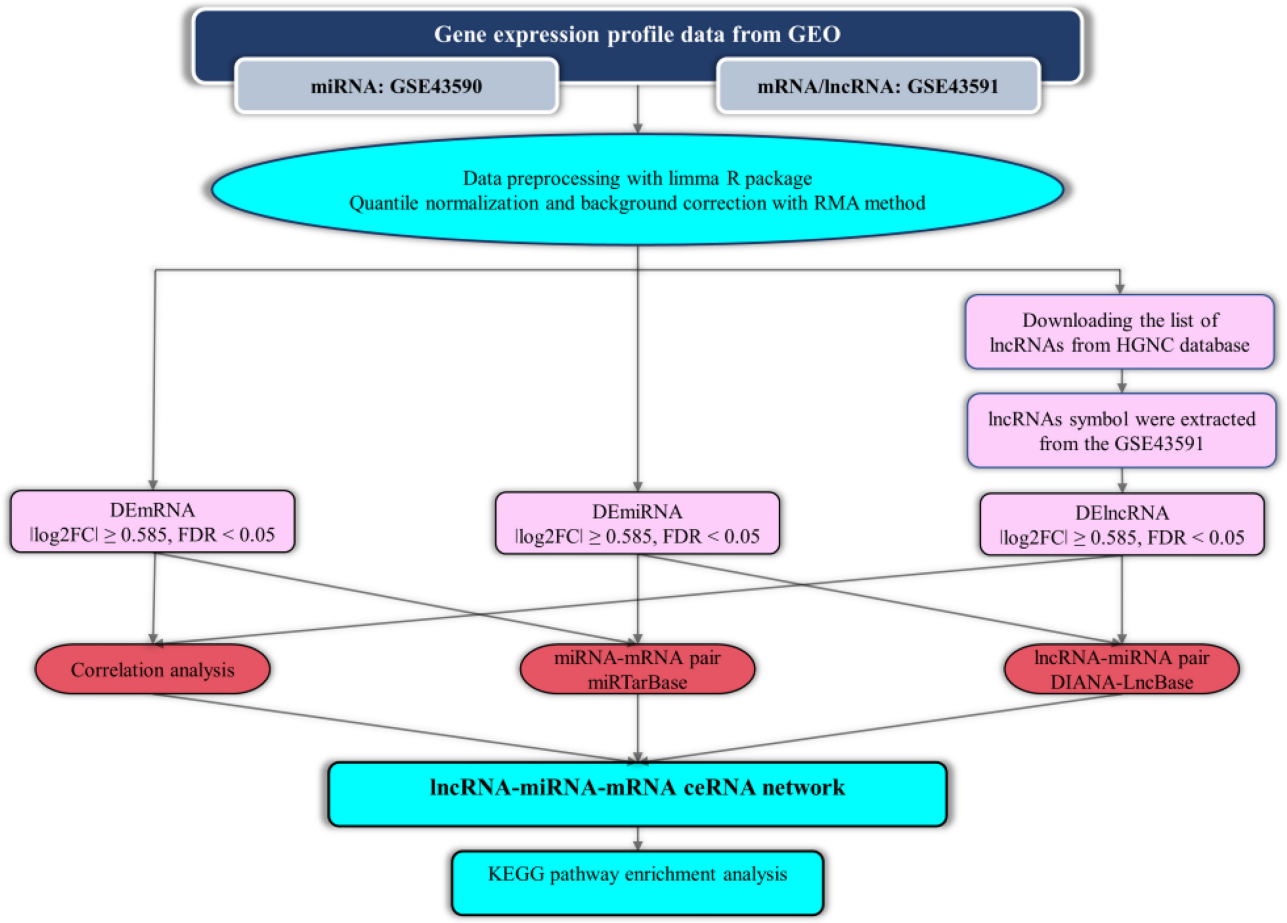
Flow chart of bioinformatics analysis.

In the present study, we utilized a system biology methodology for mining data of the two microarray datasets of peripheral blood T-cell (GSE43590 and GSE43591) from patients with RRMS and matched controls, which are SubSeries of the SuperSeries GSE43592 (11). We intended to identify differentially expressed miRNAs (DEmiRNAs), mRNAs (DEmRNAs), and lncRNAs (DElncRNAs) and construct lncRNA-associated ceRNA axes. **Figure 1** summarizes the stages performed in the bioinformatics strategy. The study protocol was approved by Ethical Committee of Shahid Beheshti University of Medical Sciences and all methods were performed in accordance with the relevant guidelines and regulations.

### Gene Expression Profile Data Collection

The miRNA profile data GSE43590 and lncRNA/mRNA profile data GSE43591 were obtained from the NCBI Gene Expression Omnibus database (GEO, https://www.ncbi.nlm.nih.gov/geo/). The platforms GPL14613 (miRNA-2) Affymetrix Multispecies miRNA-2 Array and GPL570 (HG-U133_Plus_2) Affymetrix Human Genome U133 Plus 2.0 Array were applied for GSE43590 and GSE43591 datasets, respectively. The GSE43590 included 11 peripheral blood samples from RRMS patients and nine from control subjects. The GSE43591 contained 20 peripheral blood samples, of which ten were from RRMS patients, and ten were served as controls.

### Data Preprocessing and DEmRNAs, DElncRNAs, and DEmiRNAs Identification

Two datasets were analyzed separately. The Robust Multichip Average (RMA) was employed for background correction and quantile normalization of the entire raw data files (12). The quality was assessed by the AgiMicroRna Bioconductor package (version 2.40.0). The principal component analysis (PCA) was applied for a dimensional reduction analysis (13) to find similarities between each sample group by the ggplot2 package in R software version 4.0.3. Differential gene expression analysis (DGEA) was performed between RRMS and normal samples by the linear models for microarray data (limma) R package (14) in Bioconductor (https://www.bioconductor.org/) (15). The miRNAmeConverter Bioconductor package (16) was used to convert all miRNA names to miRBase v22. The previously applied methodology wasfor QA employed to detect lncRNA probes (17). We downloaded the full list of lncRNA genes with the approved HUGO Gene Nomenclature Committee (HGNC) symbols from (https://www.genenames.org/) (18). Then, we compared the lncRNA gene list with our dataset gene symbols and chose the overlapped genes. Student t-test was utilized to detect statistically significant genes and the aberrantly expressed RNAs cut-off was set as: (1) a false discovery rate (adjusted *P*-value) < 0.05, and (2) |log2 fold change (log2FC)| ≥ 0.585. The heat map of DEmiRNAs and volcano plot of DEmRNAs/DElncRNAs were drawn using the Pheatmap (version 1.0.12) and ggplot2 packages of R.

### Protein-Protein Interaction (PPI) Network Analysis and lncRNA-Associated ceRNA Axes Construction

PPIs were identified amongst the DEmRNAs using the Search Tool for the Retrieval of Interacting Genes/Proteins (STRING, https://string-db.org/) (19). For PPI network construction, a combined score of 0.4 (medium confidence) was selected. The PPI network was depicted using Cytoscape software (version 3.8.0) (20). In addition, the Molecular Complex Detection (MCODE) cytoscape plugin (version 2.0.0) was used to select most significant module in the PPI network (21). The experimentally validated interactions between miRNAs and lncRNAs were identified using DIANA-LncBase v3 (22). *Homo Sapiens* “Species” and high “miRNA Confidence Levels” were selected as criteria for the DIANA-LncBase query. Furthermore, we acquired the interactions between miRNAs and target mRNAs from miRTarBase (23), which were supported by experimental evidence. Next, a comparison was made between the obtained mRNAs and the previously attained mRNAs. Duplicated mRNAs were then utilized for constructing the lncRNA-miRNA-mRNA axes. LncRNAs, targeted mRNAs, and the interacted miRNAs were retrieved from the ceRNA axes based on the observed opposite expression pattern between the target mRNAs and lncRNAs. The ceRNA regulatory axes were generated by the Cytoscape software.

### Correlation Analysis

We performed Pearson correlation analysis to find positive correlations between lncRNAs and mRNAs that were in the ceRNA axes. We used Hmisc (version 4.5.0) and psych (version 2.1.3) packages for the calculation of the correlations and visualization.

### Kyoto Encyclopedia of Genes and Genomes (KEGG) Pathway Enrichment Analysis

The KEGG pathway enrichment analysis was done by the Enrichr tool (24, 25) for pathway enrichment for analyzing the DEmRNAs existing in the ceRNA axes.

## Results

### DEmRNAs and DElncRNAs Identification

Background correction and normalization were performed prior to DGEA. The AgiMicroRna Bioconductor package was employed for controlling the quality of data. The spatial distribution of samples was demonstrated by a PCA plot (**Supplementary File S1**), which represents information concerning the structure of the examined data, and is helpful in finding similarities between samples. PCA showed that the samples were heterogeneous. For noise reduction, few samples [GSM1065996, GSM1065997 (two control samples), and GSM1066022 (a patient sample)] were excluded from the analysis. As it is necessary to balance between noise reduction and sample size drop, only the most obvious outliers were removed. Three samples were excluded from further analysis: two control samples from GSE43590 and a RRMS sample from GSE43591.

**Figure 2:**
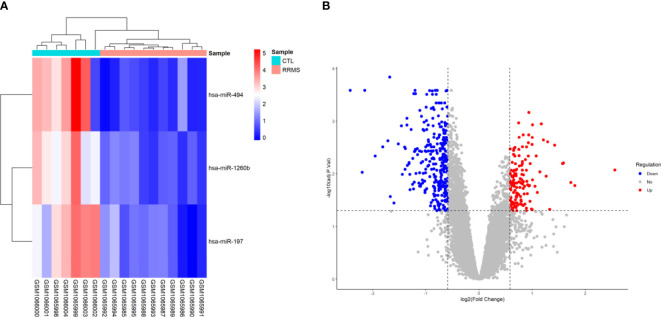
Differentially expressed genes between relapsing-remitting multiple sclerosis (RRMS) samples and control (CTL) samples. **(A)** Heatmap of the DEmiRNAs in dataset GSE43590. The normalized relative expression values are in a range between zero and five. High expressed genes are shown in red, while those expressed at low levels are blue. **(B)** Volcano plot for the DEmRNAs and DElncRNAs in dataset GSE43591. The x-axis shows the log Fold Change, and the y-axis shows the -log10 (adjusted *P*-value). Upregulated and downregulated genes are represented by red and blue dots, respectively. The gray dots represent genes with no significant difference. The DEGs were screened according to a |(log2FC)≥ 0.585 and an adjusted *P*-value < 0.05.

Based on the criteria of adjusted *P*-value < 0.05, and (2) |log2 fold change (log2FC)| ≥ 0.585, a total of three human DEmiRNAs were identified from GSE43590, all of them being were downregulated. In the GSE43591 dataset, 19 DElncRNA (12 downregulated and seven upregulated), and 467 DEmRNA (307 downregulated and 160 upregulated) were screened. A heatmap for GSE43590 and a volcano plot for GSE43591 are shown in **Figure 2**. The details of DEGs are summarized in **Supplementary File S1**.

### PPI Network Analysis and lncRNA-Associated ceRNA Axes Construction

**Figure 3:**
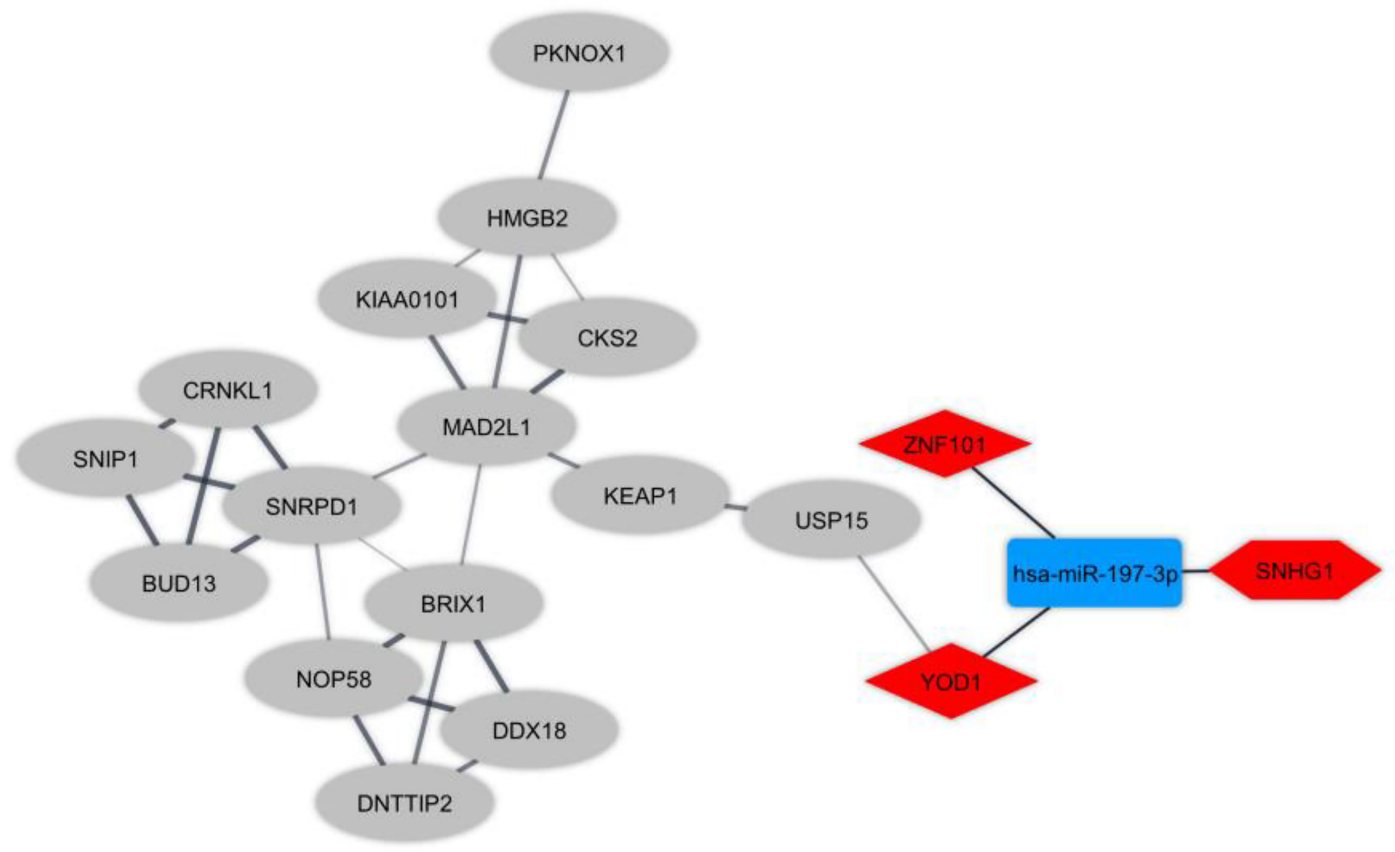
The long non-coding RNA-associated competing endogenous RNA (ceRNA) axes in T-cell in Multiple sclerosis. The red and blue nodes represent the upregulation and downregulation, respectively. The hexagon nodes and the round rectangle nodes represent the lncRNA and miRNA, respectively. The diamond nodes represent miRNAs targeted mRNAs. The ellipse nodes represent downstream connected mRNAs.

Protein interactions amongst DEmRNAs were identified (cutoff score ≥ 0.4) using the online STRING tool. We eliminated non-interacting genes from the PPI network to simplify it. Furthermore, the highly connected module was detected by plugin MCODE. To elucidate the regulatory mechanism in T-cells in RRMS, we made regulatory ceRNA axes based on DElncRNA-DEmiRNA and DEmiRNA-DEmRNA interactions which were obtained from DIANA-LncBase and mirTarBase, respectively. DELncRNAs, targeted DEmRNAs, and also the interacted DEmiRNAs were deleted from the ceRNA axes in the opposite expression pattern present between DElncRNAs and the targeted DEmRNAs. In total, one key lncRNA (*SNHG1*: small nucleolar RNA host gene 1), one key miRNA (*hsa-miR-197-3p*), and two key mRNAs (*YOD1*: YOD1 deubiquitinase and *ZNF101*: zinc finger protein 101) were identified. The ceRNA axes in T-cell in MS and the downstream connected genes are depicted in **Figure 3**.

### Correlation Analysis

**Figure 4:**
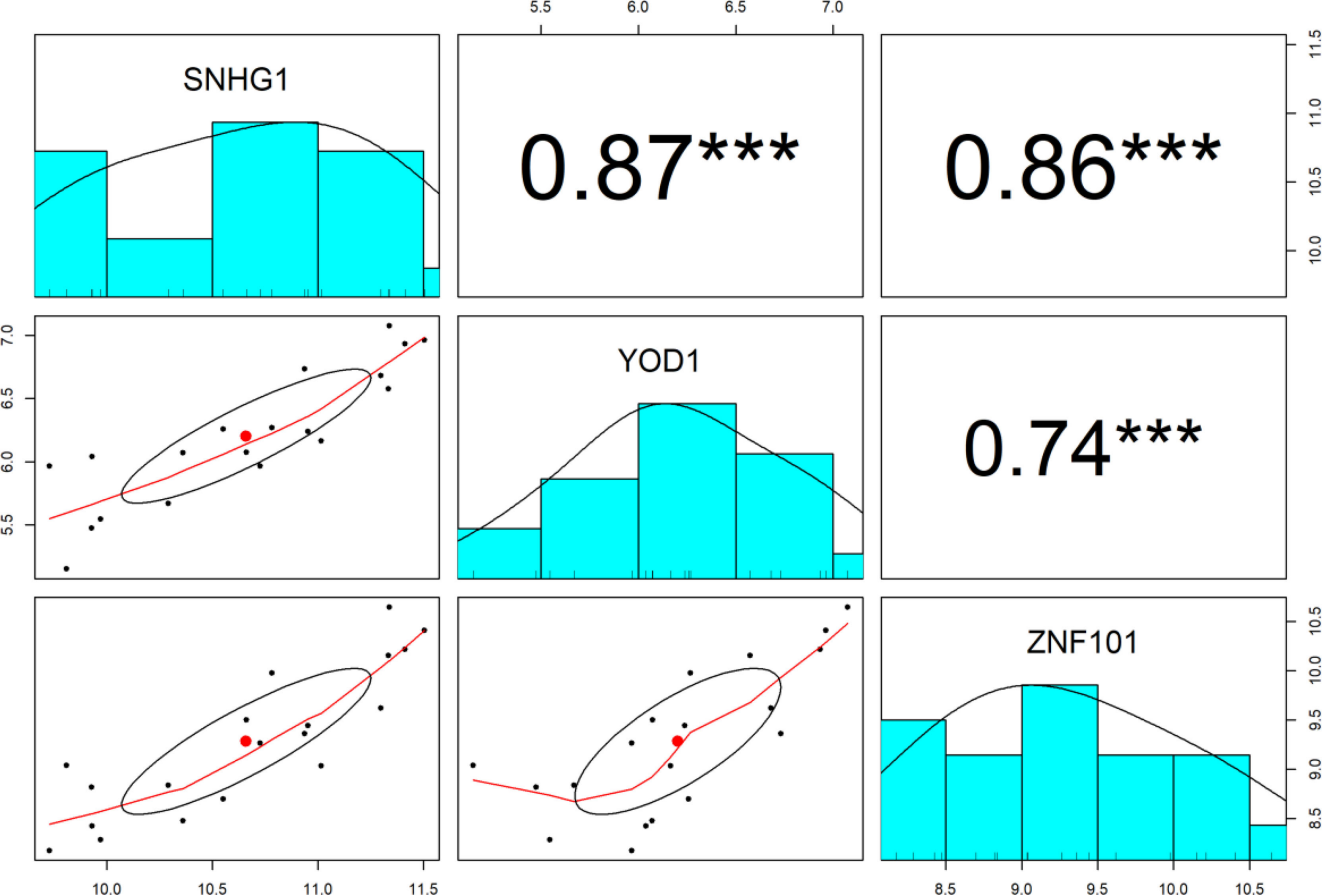
The distribution of each variable is shown on the diagonal. The lower portion of the diagonal shows bivariate scatter plots with a fitted line. On the upper part of the diagonal, the correlation coefficients plus the significance level as stars are displayed. *** is significant correlation at *P*-value < 0.001.

The Pearson correlation analysis between lncRNA *SNHG1* and target mRNAs (*YOD1* and *ZNF101*) was conducted for the verification of the hypothesis that in the ceRNA axes, mRNA expression is positively regulated by lncRNA through interaction with miRNA (**Figure 4**). The results showed that expression of *SNHG1* is positively correlated with *YOD1* (r = 0.87, *P* < 0.001) and *ZNF101* (r = 0.86, *P* < 0.001).

### KEGG Pathway Enrichment Analyses

**Figure 5:**
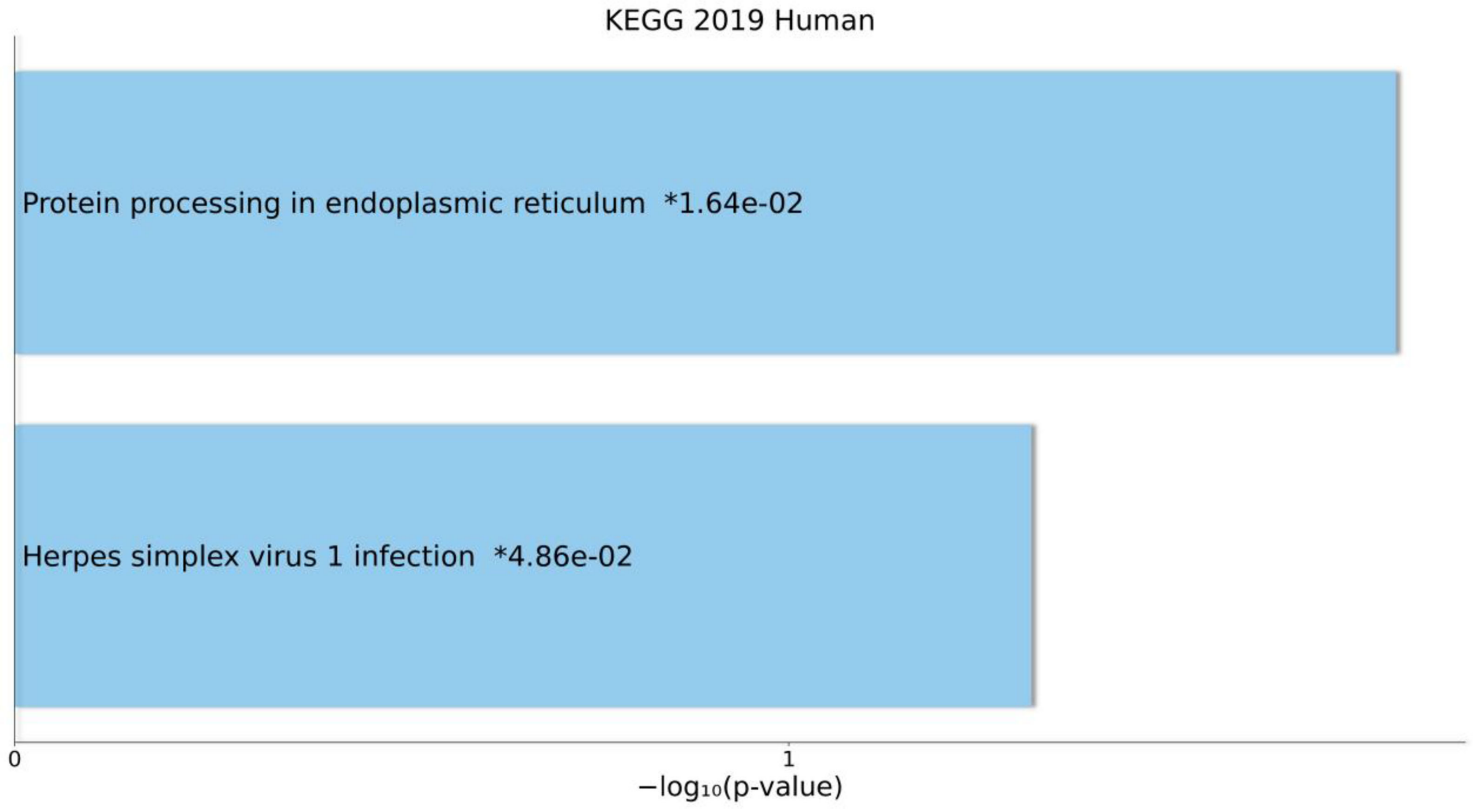
Overall results of pathway enrichment analysis using Enrichr tool. The bar chart shows the enriched pathways, along with their corresponding *P*-values. Colored bars correspond to terms with significant *P*-values (<0.05). An asterisk (*) next to a *P*-value indicates the term also has a significant adjusted *P*-value (<0.05).

The results of KEGG pathway enrichment analyses for DEmRNAs that were in the ceRNA axes are presented in **Figure 5**. The related pathways were “Protein processing in endoplasmic reticulum” and “Herpes simplex virus 1 infection”.

## Discussion

Multiple reports have shown that ceRNA regulatory axes and connected networks act actively in various developmental procedures and pathological conditions, such as tumor formation and wide-ranging brain-related disorders (26, 27). The ceRNA is expressed differently according to tissue-related, cellular, and subcellular situations. There may be a variety of ceRNAs, including lncRNAs, circRNAs, pseudogenes, as well as mRNAs, in a network. LncRNAs, one of the main types of RNAs, are detected in the ceRNA machinery and significantly contribute to cellular mechanisms in both physiological and pathological situations (28). There is currently full agreement that lncRNAs are expressed variously according to tissue, cellular types, and developmental levels. Such a specific tissue dependence, in addition to subcellular dispersions, is a clear indication of the tight regulation of lncRNAs expression (29). As denoted in the theoretic notions, the ceRNA regulatory axes connected to lncRNAs can have a critical contribution to MS pathogenicity. Up to now, research on the ceRNA axes involved in MS has been insufficient, and it is necessary to further examine the corresponding expression patterns and mechanisms in MS. In the current study, we utilized a public database to download the expression profiles of peripheral blood T-cells from RRMS patients to assess the DEmiRNAs, DElncRNAs, and DEmRNAs in RRMS and normal samples and then construct lncRNA-miRNA-mRNA regulatory axes. According to these ceRNA axes and lncRNA-mRNA co-expression relationships, we found the lncRNA-miRNA-mRNA axes consisting of one lncRNA (*SNHG1*), one miRNA (*hsa-miR-197-3p*), and two mRNAs (*YOD1* and *ZNF101*).

A higher level of lncRNA *SNHG1* was detected in patients with RRMS in comparison with the controls. *SNHG1* is a recently described lncRNA involved in the development of several tumors and other types of disorders, including Alzheimer’s disease (AD) and Parkinson’s disease (PD). In line with our result, its upregulation has been seen in *in vitro* models of PD from neurons and microglia, mouse models, and AD *in vitro* models. It is involved in the pathogenesis of AD and PD through several complementary ceRNA mechanisms (27). To our knowledge, our research is the first to report the association between *SNHG1* and MS; thus, the reported result should be validated by extra investigations.

Conversely, the level of *hsa-miR-197-3p* was lower in RRMS patients in comparison with controls. In a profiling study using microarray analysis and validation by real-time polymerase chain reaction, *hsa-miR-197-3p* was identified as a downregulated significant regulatory miRNA in T-cells in RRMS (11), consistent with our result.

The expression of *YOD1* and *ZNF101* genes was increased in RRMS cases compared with controls. *YOD1* is a highly conserved deubiquitinase-like yeast ovarian tumor domain-containing protein 1 (OTU1) related to regulating the endoplasmic reticulum (ER)-associated degradation pathway. Indeed, *YOD1* is reported to be involved in the ER stress response induced by the mislocalization of unfolded proteins in mammalian cells. *YOD1* was shown to have elevated expression levels due to different stress conditions. Moreover, *YOD1* level upregulation was reported to be induced by neurogenic proteins, causing Huntington’s disease and PD. The deubiquitinase YOD1 was proposed to contribute in the pathogenicity of neurodegenerative diseases by reducing ubiquitination of abnormal proteins and their degradation (30). Our result is in line with these findings. *ZNF101* was another gene in the ceRNA axes. Zinc finger proteins, including ZNF101 interact with nucleic acids and have lots of crucial activities, particularly regulation of transcription (31). In line with our result, a previous study reported that single nucleotide polymorphism rs1064395 in neurocan (*NCAN*) gene is associated with the upregulated expression level of *ZNF101* (32).

In the current study, KEGG pathway enrichment analysis also was conducted. The results showed that DEmRNAs that were in the ceRNA axes were enriched in “Protein processing in endoplasmic reticulum” and “Herpes simplex virus 1 infection” pathways, respectively. The unfolded protein response (UPR) happens to respond to ER stress resulting from the accretion of unfolded or misfolded proteins in the ER. The cytoprotective activities are promoted by the UPR to amend ER stress, but the influenced cells become apoptotic due to the UPR as a result of unresolved ER stress. The UPR is a central attribute of various disorders in humans, e.g., MS (33). Furthermore, an association between herpes simplex virus 1 (HSV-1) infection and demyelination has been reported in previous studies. Nonetheless, it is not certain whether HSV-1 is involved in MS etiology. Viruses, specifically HSV-1, may act as a risk factor for MS progression rather than a causative agent. This neurotropic pathogenic agent may mediate several molecular procedures (34).

There are some limitations in our study. Firstly, multiple parameters, including different methodologies, sample preparation, patient characteristics, platforms, and analyzing data, might influence the gene expression patterns. Secondly, a small sample size can cause low statistical power. Lastly, the present findings need to be validated by confirmative experimental approaches as well as re-analysis of microarray gene expression profiles.

## Conclusion

In conclusion, lncRNA *SNHG1* can serve as a ceRNA to regulate the expression of *YOD1* and *ZNF101* and downstream connected genes in T-cells in RRMS patients *via* sponging *hsa-miR-197-3p*. In our investigation, potential research targets are provided to examine molecular mechanisms that underpin the pathogenicity of MS.

## Data Availability Statement

The raw data supporting the conclusions of this article will be made available by the authors, without undue reservation.

## Author Contributions

MT, HS, BH, and MR wrote the manuscript and revised it. SG-F, NA, ZS, MAB, and MRA performed the bioinformatic analysis and collected the information. All authors contributed to the article and approved the submitted version.

## Funding

The research protocol was approved & supported by Molecular Medicine Research Center, Tabriz University of Medical Sciences (grant number: 67290).

## Conflict of Interest

The authors declare that the research was conducted in the absence of any commercial or financial relationships that could be construed as a potential conflict of interest.

## Publisher’s Note

All claims expressed in this article are solely those of the authors and do not necessarily represent those of their affiliated organizations, or those of the publisher, the editors and the reviewers. Any product that may be evaluated in this article, or claim that may be made by its manufacturer, is not guaranteed or endorsed by the publisher.
