# Supplementary material for: Long Non-Coding RNA- Associated Competing Endogenous RNA Axes in T-Cells in Multiple Sclerosis
Source: Front Immunol. 2021 Dec 8;12:770679. doi: 10.3389/fimmu.2021.770679 (PMC8696673; doi:10.3389/fimmu.2021.770679)
Supplement: Supplementary file 1 [file DataSheet_1.docx]

**
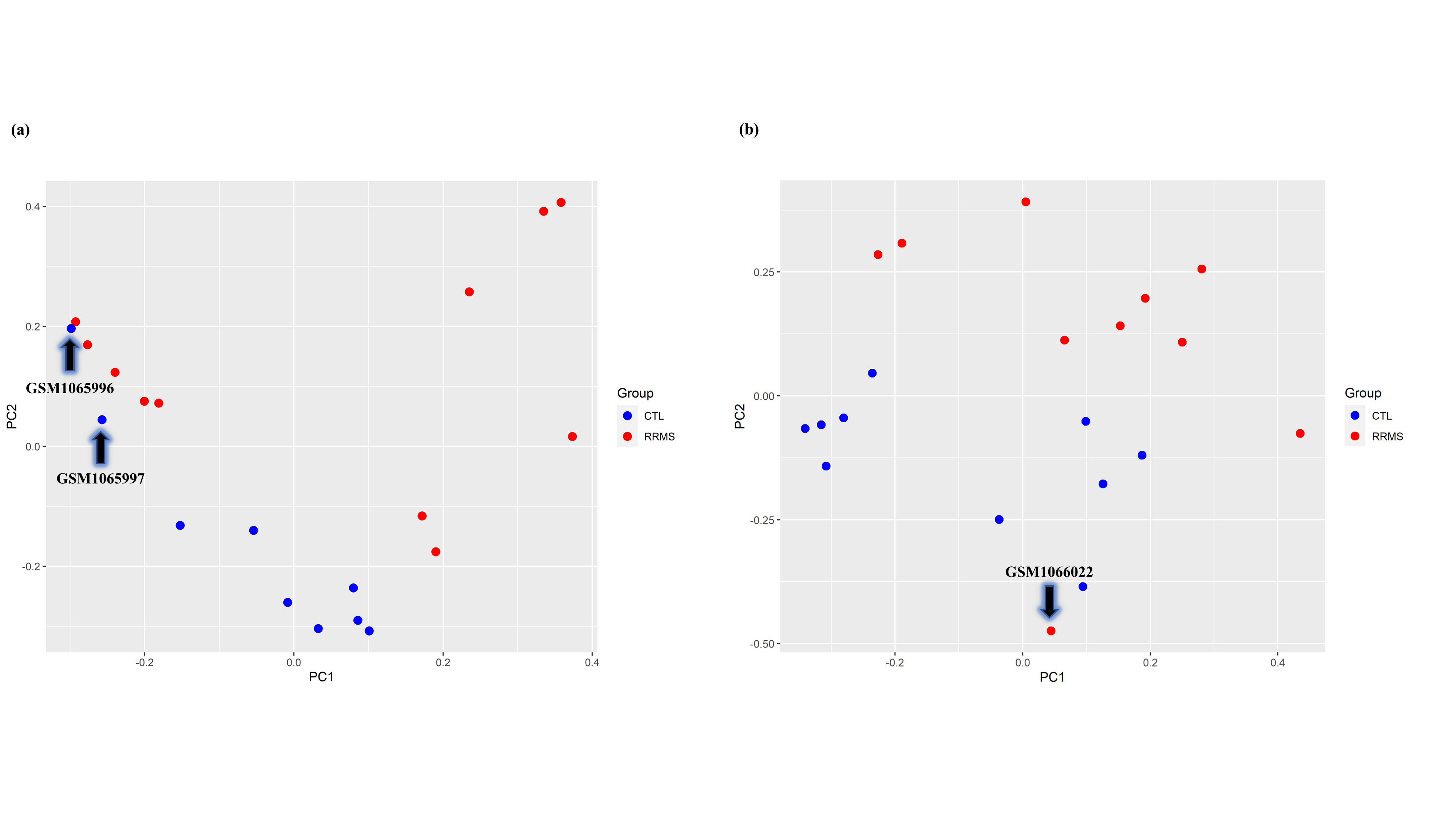
Figure S1** Principal component analysis (PCA) for the **(a)** GSE43590 and **(b)** GSE43591 studies. GSM1065996, GSM1065997 (two control samples), and GSM1066022 (a patient sample) were removed from further analysis in order to their wrong spatial enrichment. CTL, control; RRMS, relapsing-remitting multiple sclerosis.

| Table S1 Differentially expressed miRNAs with \|log2 fold change \| ≥ 0.585 and adjusted P value < 0.05 | | | | | | |
| --- | --- | --- | --- | --- | --- | --- |
| PROBEID | logFC | AveExpr | t | P.Value | adj.P.Val | B |
| hsa-miR-494_st | -2.84818 | 1.564574 | -6.76976 | 2.11E-06 | 0.002298 | 4.85548 |
| hsa-miR-197_st | -2.31592 | 1.74478 | -7.1868 | 9.33E-07 | 0.001208 | 5.552071 |
| hsa-miR-1260b_st | -2.09321 | 1.599847 | -9.31894 | 2.07E-08 | 3.90E-05 | 8.666976 |

| Table S2 Differentially expressed mRNAs and lncRNAs with \|log2 fold change \| ≥ 0.585 and adjusted P value < 0.05 | | | | | | | |
| --- | --- | --- | --- | --- | --- | --- | --- |
| PROBEID | logFC | AveExpr | t | P.Value | adj.P.Val | B | SYMBOL |
| 1552634_a_at | 1.079756 | 9.282489 | 5.091326 | 4.49E-05 | 0.00458 | 2.159435 | ZNF101 |
| 1552703_s_at | 0.725573 | 8.050406 | 3.884788 | 0.000824 | 0.023301 | -0.58557 | CARD16//CASP1 |
| 1553099_at | 0.942438 | 7.0019 | 6.729079 | 1.03E-06 | 0.000679 | 5.710212 | TIGD1 |
| 1554036_at | 1.102594 | 6.986886 | 3.909159 | 0.000777 | 0.022502 | -0.53035 | ZBTB24 |
| 1554057_at | 0.837401 | 6.767507 | 4.01227 | 0.000606 | 0.018954 | -0.29631 | ASH1L-AS1 |
| 1554089_s_at | 1.011693 | 8.70431 | 6.25281 | 2.99E-06 | 0.001168 | 4.712334 | SBDSP1//SBDS |
| 1554240_a_at | -0.59652 | 10.30945 | -3.39963 | 0.002631 | 0.046681 | -1.67158 | ITGAL |
| 1555486_a_at | -1.19068 | 7.813208 | -5.31108 | 2.66E-05 | 0.003438 | 2.653188 | PRR5L |
| 1556033_at | -0.64441 | 7.377856 | -5.70866 | 1.05E-05 | 0.002193 | 3.535004 | LINC01138 |
| 1556180_at | -0.7508 | 6.629789 | -6.28974 | 2.75E-06 | 0.001123 | 4.790857 | LINC00847 |
| 1560228_at | -0.85207 | 6.026574 | -5.76939 | 9.08E-06 | 0.002025 | 3.668187 | SNAI3 |
| 1564139_at | -2.14987 | 8.019379 | -7.85262 | 9.32E-08 | 0.00026 | 7.93009 | A2M-AS1 |
| 1568780_at | -0.78598 | 6.015704 | -3.63148 | 0.001515 | 0.034084 | -1.15622 | LOC729732 |
| 1569940_at | -0.63015 | 5.55807 | -4.71962 | 0.00011 | 0.007117 | 1.316717 | SLC6A16 |
| 200617_at | -0.87879 | 7.711339 | -7.46028 | 2.11E-07 | 0.000309 | 7.176979 | MLEC |
| 200790_at | 0.624022 | 9.802087 | 4.168491 | 0.000415 | 0.015631 | 0.059365 | ODC1 |
| 200799_at | -1.24928 | 8.375973 | -4.98679 | 5.77E-05 | 0.005116 | 1.923278 | HSPA1A//HSPA1B |
| 200800_s_at | -0.88454 | 7.056286 | -5.24095 | 3.15E-05 | 0.00377 | 2.496046 | HSPA1A//HSPA1B//HSPA1L |
| 200907_s_at | -0.83476 | 5.160767 | -4.01268 | 0.000605 | 0.018954 | -0.29537 | PALLD |
| 200931_s_at | -0.83905 | 9.168671 | -4.21826 | 0.000368 | 0.014519 | 0.172855 | VCL |
| 201061_s_at | -0.62832 | 9.608377 | -3.64078 | 0.001481 | 0.033542 | -1.13539 | STOM |
| 201502_s_at | 0.941081 | 11.3806 | 5.857636 | 7.40E-06 | 0.001813 | 3.860977 | NFKBIA |
| 201656_at | 0.641788 | 9.063389 | 3.350667 | 0.002954 | 0.049861 | -1.77931 | ITGA6 |
| 201695_s_at | -0.81467 | 7.767681 | -4.78252 | 9.42E-05 | 0.006475 | 1.459848 | PNP |
| 201703_s_at | -0.68528 | 7.679658 | -5.5014 | 1.70E-05 | 0.002774 | 3.07732 | PPP1R10 |
| 201739_at | 1.42826 | 6.802134 | 5.471193 | 1.83E-05 | 0.002863 | 3.010244 | SGK1 |
| 201745_at | 1.010758 | 7.442965 | 4.293627 | 0.000307 | 0.012999 | 0.344843 | TWF1 |
| 201818_at | -0.95462 | 9.217959 | -5.46362 | 1.86E-05 | 0.002873 | 2.993413 | LPCAT1 |
| 201960_s_at | 0.687951 | 9.935376 | 3.602274 | 0.001624 | 0.035596 | -1.22157 | MYCBP2 |
| 201991_s_at | 0.675214 | 9.511921 | 4.197611 | 0.000387 | 0.014966 | 0.125763 | KIF5B |
| 202220_at | -0.62738 | 8.995771 | -5.56464 | 1.47E-05 | 0.002619 | 3.217467 | KHDC4 |
| 202304_at | 0.666979 | 7.967124 | 3.566019 | 0.001771 | 0.03768 | -1.30252 | FNDC3A |
| 202361_at | -0.71064 | 8.838617 | -6.13895 | 3.87E-06 | 0.001271 | 4.469115 | SEC24C |
| 202388_at | 1.576194 | 8.472626 | 4.799945 | 9.04E-05 | 0.00638 | 1.499457 | RGS2 |
| 202417_at | -0.90062 | 7.283373 | -6.93416 | 6.55E-07 | 0.000571 | 6.12977 | KEAP1 |
| 202441_at | -0.61329 | 7.290239 | -4.94707 | 6.35E-05 | 0.005323 | 1.833335 | ERLIN1 |
| 202458_at | -1.34883 | 6.844425 | -4.73993 | 0.000104 | 0.00694 | 1.362958 | PRSS23 |
| 202467_s_at | 0.655992 | 9.064219 | 3.910017 | 0.000775 | 0.022502 | -0.52841 | COPS2 |
| 202503_s_at | 0.788758 | 5.912961 | 3.778881 | 0.001063 | 0.026933 | -0.82493 | PCLAF |
| 202542_s_at | 0.613227 | 8.985058 | 4.585283 | 0.000152 | 0.008564 | 1.010578 | AIMP1 |
| 202595_s_at | 0.593822 | 10.76997 | 3.676468 | 0.00136 | 0.031682 | -1.05537 | LEPROTL1 |
| 202616_s_at | -0.60707 | 7.275766 | -7.04153 | 5.18E-07 | 0.000538 | 6.346905 | MECP2 |
| 202623_at | 0.626611 | 8.705347 | 4.38922 | 0.000243 | 0.011224 | 0.56308 | EAPP |
| 202644_s_at | 1.17665 | 11.296 | 6.284538 | 2.78E-06 | 0.001123 | 4.7798 | TNFAIP3 |
| 202677_at | 0.668135 | 8.413238 | 4.085569 | 0.000508 | 0.017194 | -0.12956 | RASA1 |
| 202679_at | -0.70484 | 7.483667 | -4.43026 | 0.00022 | 0.010566 | 0.656792 | NPC1 |
| 202690_s_at | 0.784167 | 8.370783 | 3.575871 | 0.00173 | 0.037202 | -1.28054 | SNRPD1 |
| 202708_s_at | -0.86061 | 6.71345 | -4.08189 | 0.000512 | 0.017248 | -0.13794 | H2BC21 |
| 202710_at | -0.63687 | 6.339409 | -3.95907 | 0.000689 | 0.020721 | -0.41714 | BET1 |
| 202716_at | -0.85336 | 8.102519 | -3.86187 | 0.000871 | 0.0241 | -0.63745 | PTPN1 |
| 202776_at | 0.678665 | 8.693359 | 4.470495 | 0.0002 | 0.009979 | 0.748637 | DNTTIP2 |
| 202832_at | 0.666399 | 8.154077 | 3.553738 | 0.001824 | 0.03827 | -1.32991 | GCC2 |
| 202838_at | -0.62298 | 7.000612 | -3.86414 | 0.000866 | 0.024043 | -0.63231 | FUCA1 |
| 202842_s_at | 0.618168 | 8.29281 | 3.421766 | 0.002497 | 0.045356 | -1.62274 | DNAJB9 |
| 202887_s_at | 0.788032 | 9.771177 | 4.209803 | 0.000376 | 0.014686 | 0.153571 | DDIT4 |
| 202973_x_at | 1.806711 | 7.496783 | 4.115479 | 0.000472 | 0.016692 | -0.06145 | FAM13A |
| 203044_at | -0.64173 | 7.616662 | -3.65296 | 0.001439 | 0.03292 | -1.1081 | CHSY1 |
| 203159_at | -0.67106 | 8.563486 | -6.03665 | 4.90E-06 | 0.001437 | 4.249118 | GLS |
| 203252_at | -0.71521 | 7.782156 | -4.94724 | 6.34E-05 | 0.005323 | 1.833717 | CDK2AP2 |
| 203317_at | -0.60026 | 8.472503 | -4.94306 | 6.41E-05 | 0.005333 | 1.824253 | PSD4 |
| 203344_s_at | 0.644363 | 4.035054 | 4.233033 | 0.000355 | 0.014267 | 0.206564 | RBBP8 |
| 203362_s_at | 0.797571 | 5.604622 | 3.688914 | 0.00132 | 0.031246 | -1.02743 | MAD2L1 |
| 203471_s_at | -1.25332 | 8.778898 | -4.1615 | 0.000422 | 0.015826 | 0.043416 | PLEK |
| 203544_s_at | 0.59673 | 7.425805 | 4.215817 | 0.00037 | 0.014552 | 0.16729 | STAM |
| 203562_at | -0.83929 | 6.094498 | -3.49533 | 0.002096 | 0.041271 | -1.45983 | FEZ1 |
| 203595_s_at | 0.991809 | 7.125648 | 3.403994 | 0.002604 | 0.04653 | -1.66196 | IFIT5 |
| 203713_s_at | -0.62288 | 6.72403 | -4.06366 | 0.000535 | 0.017653 | -0.17942 | LLGL2 |
| 203725_at | 0.760928 | 7.200671 | 5.78855 | 8.69E-06 | 0.002025 | 3.71013 | GADD45A |
| 203823_at | -0.95735 | 7.758206 | -6.22508 | 3.18E-06 | 0.00118 | 4.653259 | RGS3 |
| 203846_at | -0.66089 | 6.988429 | -4.53393 | 0.000172 | 0.009149 | 0.893425 | TRIM32 |
| 203856_at | 0.647563 | 7.030868 | 4.371879 | 0.000254 | 0.011481 | 0.523488 | VRK1 |
| 203956_at | -0.70584 | 7.975629 | -4.59976 | 0.000146 | 0.008444 | 1.043603 | MORC2 |
| 204015_s_at | 0.59688 | 5.961044 | 3.768245 | 0.001091 | 0.027314 | -0.84891 | DUSP4 |
| 204066_s_at | -1.35833 | 6.442193 | -6.51334 | 1.66E-06 | 0.000878 | 5.262191 | AGAP1 |
| 204079_at | -0.85917 | 8.412082 | -4.77594 | 9.57E-05 | 0.006525 | 1.444882 | TPST2 |
| 204103_at | -1.09305 | 8.666512 | -4.16286 | 0.000421 | 0.015801 | 0.046527 | CCL4 |
| 204137_at | -0.8494 | 5.871696 | -4.45042 | 0.00021 | 0.010266 | 0.702815 | GPR137B |
| 204170_s_at | -1.01847 | 4.61519 | -3.90518 | 0.000785 | 0.02263 | -0.53936 | CKS2 |
| 204198_s_at | -0.70075 | 11.14363 | -4.95107 | 6.29E-05 | 0.005323 | 1.842403 | RUNX3 |
| 204205_at | -0.75096 | 9.777207 | -3.39775 | 0.002643 | 0.046813 | -1.67573 | APOBEC3G |
| 204244_s_at | 0.877937 | 5.899 | 4.939564 | 6.46E-05 | 0.005357 | 1.816335 | DBF4 |
| 204346_s_at | -0.9555 | 8.548367 | -5.68173 | 1.11E-05 | 0.002276 | 3.475786 | RASSF1 |
| 204436_at | -0.75341 | 7.67026 | -7.14711 | 4.13E-07 | 0.00045 | 6.558723 | PLEKHO2 |
| 204439_at | 2.556535 | 7.185831 | 4.595765 | 0.000148 | 0.008481 | 1.034485 | IFI44L |
| 204440_at | 0.656986 | 6.836033 | 5.287937 | 2.81E-05 | 0.003594 | 2.601379 | CD83 |
| 204512_at | 0.599885 | 6.67394 | 3.394445 | 0.002663 | 0.047028 | -1.68301 | HIVEP1 |
| 204655_at | -1.00452 | 11.84295 | -5.39967 | 2.16E-05 | 0.003079 | 2.85105 | CCL5 |
| 204735_at | -0.63438 | 6.817988 | -3.86792 | 0.000858 | 0.023964 | -0.62375 | PDE4A |
| 204749_at | 0.964824 | 5.958314 | 3.649587 | 0.00145 | 0.033082 | -1.11566 | NAP1L3 |
| 204790_at | -0.77448 | 7.153478 | -3.81535 | 0.000974 | 0.025522 | -0.74261 | SMAD7 |
| 204811_s_at | -0.83331 | 7.298357 | -3.94987 | 0.000704 | 0.021041 | -0.43802 | CACNA2D2 |
| 204852_s_at | -0.7886 | 8.51644 | -7.91394 | 8.21E-08 | 0.00026 | 8.045617 | PTPN7 |
| 204917_s_at | 0.664143 | 8.166975 | 4.127156 | 0.000459 | 0.016603 | -0.03484 | MLLT3 |
| 204923_at | -0.8074 | 10.24901 | -7.18048 | 3.84E-07 | 0.00045 | 6.625327 | SASH3 |
| 204971_at | 0.898163 | 5.566956 | 3.431127 | 0.002442 | 0.044818 | -1.60206 | CSTA |
| 205013_s_at | -0.68085 | 7.86554 | -5.15335 | 3.88E-05 | 0.004269 | 2.29918 | ADORA2A//SPECC1L-ADORA2A |
| 205233_s_at | -0.71543 | 6.204147 | -4.71951 | 0.00011 | 0.007117 | 1.316473 | PAFAH2 |
| 205239_at | 0.68187 | 4.398356 | 5.381449 | 2.26E-05 | 0.003153 | 2.810413 | AREG |
| 205281_s_at | 0.809091 | 5.184442 | 4.744201 | 0.000103 | 0.00689 | 1.372669 | PIGA |
| 205291_at | -0.84923 | 9.875211 | -4.63667 | 0.000134 | 0.008043 | 1.127761 | IL2RB |
| 205315_s_at | -0.64436 | 7.857475 | -6.37365 | 2.27E-06 | 0.001055 | 4.968544 | SNTB2 |
| 205417_s_at | -0.60927 | 7.418845 | -5.67269 | 1.14E-05 | 0.002297 | 3.455903 | DAG1 |
| 205484_at | -0.66685 | 8.749913 | -5.78043 | 8.85E-06 | 0.002025 | 3.692357 | SIT1 |
| 205488_at | -0.8413 | 10.0364 | -4.05728 | 0.000543 | 0.01782 | -0.19395 | GZMA |
| 205495_s_at | -1.81278 | 10.54398 | -5.40263 | 2.15E-05 | 0.003079 | 2.857645 | GNLY |
| 205633_s_at | -0.78774 | 6.857098 | -5.5374 | 1.56E-05 | 0.002703 | 3.157142 | ALAS1 |
| 205639_at | -0.91665 | 7.16503 | -5.5075 | 1.68E-05 | 0.002774 | 3.090861 | AOAH |
| 205758_at | -1.03442 | 10.60842 | -6.38799 | 2.20E-06 | 0.001043 | 4.998819 | CD8A |
| 205786_s_at | -1.46168 | 8.218248 | -6.17917 | 3.53E-06 | 0.001221 | 4.555232 | ITGAM |
| 205898_at | -2.19661 | 9.417083 | -4.51886 | 0.000178 | 0.009328 | 0.859026 | CX3CR1 |
| 206082_at | -1.05502 | 8.129459 | -6.04471 | 4.81E-06 | 0.001437 | 4.266514 | HCP5 |
| 206170_at | -1.39239 | 7.243513 | -4.61726 | 0.00014 | 0.008225 | 1.08351 | ADRB2 |
| 206267_s_at | -0.78858 | 7.867484 | -5.18306 | 3.61E-05 | 0.004101 | 2.36603 | MATK |
| 206366_x_at | -0.75686 | 7.011287 | -3.49601 | 0.002093 | 0.041271 | -1.45834 | XCL1 |
| 206518_s_at | -0.67109 | 5.569118 | -4.14032 | 0.000445 | 0.016249 | -0.00485 | RGS9 |
| 206589_at | -0.719 | 7.355452 | -4.36942 | 0.000255 | 0.011507 | 0.517865 | GFI1 |
| 206656_s_at | -0.68597 | 9.837593 | -5.41802 | 2.07E-05 | 0.003069 | 2.891934 | APMAP |
| 206695_x_at | 0.715048 | 6.152186 | 4.62407 | 0.000138 | 0.008195 | 1.099026 | ZNF43 |
| 206777_s_at | 0.703845 | 5.69755 | 4.41239 | 0.00023 | 0.010865 | 0.615981 | CRYBB2//CRYBB2P1 |
| 206914_at | -1.20476 | 6.028869 | -8.19835 | 4.60E-08 | 0.00026 | 8.573846 | CRTAM |
| 206978_at | -1.40146 | 6.932289 | -5.39178 | 2.20E-05 | 0.003117 | 2.833459 | CCR2 |
| 206991_s_at | -1.08296 | 8.90402 | -5.23307 | 3.21E-05 | 0.003774 | 2.47836 | CCR5 |
| 207338_s_at | -0.70182 | 6.880166 | -3.81451 | 0.000976 | 0.025522 | -0.74452 | ZNF200 |
| 207460_at | -0.65071 | 9.851498 | -5.65105 | 1.20E-05 | 0.002372 | 3.408255 | GZMM |
| 207643_s_at | -0.93124 | 7.276567 | -7.77972 | 1.08E-07 | 0.000262 | 7.791968 | TNFRSF1A |
| 207907_at | -0.95234 | 5.919406 | -5.25962 | 3.01E-05 | 0.003688 | 2.537915 | TNFSF14 |
| 207979_s_at | -0.66413 | 10.03955 | -4.10527 | 0.000484 | 0.016883 | -0.08469 | CD8B |
| 208078_s_at | 1.003968 | 6.59871 | 5.508488 | 1.67E-05 | 0.002774 | 3.093054 | ZEB1//SIK1 |
| 208093_s_at | -0.68038 | 8.08008 | -5.69372 | 1.08E-05 | 0.002249 | 3.50217 | NDEL1 |
| 208438_s_at | -1.13412 | 8.324822 | -4.19205 | 0.000392 | 0.014968 | 0.113083 | FGR |
| 208490_x_at | -0.59727 | 5.966487 | -3.35001 | 0.002959 | 0.049861 | -1.78075 | H2BC7//H2BC8//H2BC6//H2BC10//H2BC4 |
| 208774_at | -0.63535 | 9.579227 | -6.19062 | 3.44E-06 | 0.001221 | 4.579707 | CSNK1D |
| 208808_s_at | 0.822157 | 9.979505 | 4.871885 | 7.60E-05 | 0.005857 | 1.662862 | HMGB2 |
| 208895_s_at | 0.763292 | 8.446681 | 4.718203 | 0.00011 | 0.007118 | 1.313494 | DDX18 |
| 208916_at | -0.59606 | 6.426063 | -4.57817 | 0.000154 | 0.008566 | 0.994345 | SLC1A5 |
| 209067_s_at | 0.663645 | 10.78752 | 5.345734 | 2.45E-05 | 0.003324 | 2.730671 | HNRNPDL |
| 209096_at | 0.651198 | 7.96974 | 3.448525 | 0.002343 | 0.043851 | -1.56358 | UBE2V2 |
| 209156_s_at | -1.30467 | 7.692247 | -4.45223 | 0.000209 | 0.010247 | 0.706938 | COL6A2 |
| 209181_s_at | 0.757869 | 9.552467 | 4.236939 | 0.000352 | 0.014182 | 0.215475 | RABGGTB//SNORD45B//SNORD45A//SNORD45C |
| 209185_s_at | 0.714826 | 8.828517 | 4.045361 | 0.000559 | 0.018087 | -0.22106 | IRS2 |
| 209197_at | -0.75627 | 7.199442 | -4.02047 | 0.000594 | 0.018826 | -0.27767 | SYT11 |
| 209286_at | -0.69166 | 8.1832 | -4.62446 | 0.000138 | 0.008195 | 1.09991 | CDC42EP3 |
| 209308_s_at | 0.698388 | 8.169353 | 4.577687 | 0.000154 | 0.008566 | 0.993253 | BNIP2 |
| 209312_x_at | -0.59876 | 9.809036 | -3.65438 | 0.001434 | 0.032877 | -1.10491 | HLA-DRB1//HLA-DQB1//HLA-DRB4//HLA-DRB5 |
| 209584_x_at | -0.64788 | 7.863908 | -5.12969 | 4.10E-05 | 0.004362 | 2.245905 | APOBEC3C |
| 209795_at | 1.296505 | 9.999473 | 5.610402 | 1.32E-05 | 0.002474 | 3.318603 | CD69 |
| 209813_x_at | -1.06142 | 10.25903 | -4.87659 | 7.52E-05 | 0.005842 | 1.67354 | TRGV9//TRGC2//TARP |
| 209815_at | -0.8342 | 7.660326 | -4.36765 | 0.000257 | 0.011533 | 0.513833 | PTCH1 |
| 209883_at | -0.72653 | 5.333757 | -4.18776 | 0.000396 | 0.015032 | 0.103299 | COLGALT2 |
| 209933_s_at | -1.11935 | 8.62331 | -4.50554 | 0.000184 | 0.009533 | 0.828641 | CD300A |
| 210028_s_at | 0.619684 | 6.940665 | 3.576374 | 0.001728 | 0.037195 | -1.27942 | ORC3 |
| 210054_at | 0.967782 | 7.442088 | 4.796832 | 9.10E-05 | 0.006393 | 1.49238 | HAUS3 |
| 210140_at | -1.15708 | 10.14712 | -5.13667 | 4.03E-05 | 0.004333 | 2.261624 | CST7 |
| 210288_at | -0.98492 | 8.562134 | -4.00762 | 0.000613 | 0.019087 | -0.30689 | KLRG1 |
| 210321_at | -1.66966 | 10.55889 | -3.77674 | 0.001069 | 0.026934 | -0.82976 | GZMH |
| 210387_at | -2.42354 | 5.289232 | -8.01673 | 6.65E-08 | 0.00026 | 8.237974 | NCALD//H2BC8//H2BC11 |
| 210538_s_at | 0.837923 | 8.550015 | 5.877487 | 7.07E-06 | 0.001813 | 3.904214 | BIRC3 |
| 210606_x_at | -1.23254 | 8.644601 | -3.99883 | 0.000626 | 0.019276 | -0.32685 | KLRD1 |
| 210681_s_at | 0.898336 | 8.332705 | 4.579779 | 0.000154 | 0.008566 | 0.998024 | USP15//MIR6125 |
| 210785_s_at | -0.74713 | 8.272975 | -4.19204 | 0.000392 | 0.014968 | 0.113056 | THEMIS2 |
| 210865_at | -0.86963 | 5.419737 | -3.67879 | 0.001352 | 0.031675 | -1.05015 | FASLG |
| 210970_s_at | 0.623289 | 7.002982 | 4.406964 | 0.000233 | 0.010938 | 0.603593 | IBTK |
| 211423_s_at | 0.606213 | 6.863632 | 3.976273 | 0.000661 | 0.020128 | -0.37809 | SC5D |
| 211429_s_at | 1.006483 | 6.341451 | 3.378629 | 0.002765 | 0.047974 | -1.71784 | SERPINA1 |
| 211597_s_at | -1.38539 | 9.144132 | -5.34962 | 2.43E-05 | 0.003315 | 2.739356 | HOPX |
| 211685_s_at | -0.80521 | 7.533279 | -5.28744 | 2.82E-05 | 0.003594 | 2.600259 | NCALD |
| 211999_at | 0.752248 | 11.84451 | 6.348344 | 2.41E-06 | 0.001071 | 4.915056 | H3-3A//H3-3B//MIR4738 |
| 212070_at | -1.9534 | 9.722086 | -5.08823 | 4.53E-05 | 0.004592 | 2.152446 | ADGRG1 |
| 212135_s_at | -0.62356 | 9.208764 | -4.09586 | 0.000495 | 0.017135 | -0.10612 | ATP2B4 |
| 212180_at | -0.76528 | 8.778837 | -6.60933 | 1.34E-06 | 0.00079 | 5.462352 | CRKL |
| 212195_at | 0.894816 | 9.768195 | 3.348663 | 0.002968 | 0.049921 | -1.78371 | IL6ST |
| 212241_at | 0.630661 | 7.229298 | 4.845576 | 8.10E-05 | 0.006046 | 1.603135 | POLR2M//GCOM1//MYZAP |
| 212374_at | 0.822824 | 6.976917 | 5.418066 | 2.07E-05 | 0.003069 | 2.892042 | FEM1B |
| 212407_at | -0.69038 | 7.209857 | -4.24489 | 0.000345 | 0.014018 | 0.233627 | EEF1AKNMT |
| 212408_at | -0.61096 | 8.940306 | -5.05799 | 4.87E-05 | 0.004757 | 2.0842 | TOR1AIP1 |
| 212434_at | 0.68348 | 8.112038 | 4.474295 | 0.000198 | 0.009958 | 0.757312 | GRPEL1 |
| 212443_at | -0.84541 | 8.651412 | -3.84119 | 0.000915 | 0.024889 | -0.68422 | NBEAL2 |
| 212467_at | 0.663098 | 7.240215 | 4.412987 | 0.00023 | 0.010865 | 0.617345 | DNAJC13 |
| 212473_s_at | -0.79706 | 7.729438 | -4.58579 | 0.000151 | 0.008564 | 1.011737 | MICAL2 |
| 212509_s_at | -1.19877 | 8.76553 | -5.31507 | 2.64E-05 | 0.003438 | 2.662105 | MXRA7 |
| 212579_at | 0.723696 | 9.139333 | 3.59965 | 0.001634 | 0.035676 | -1.22743 | SMCHD1 |
| 212614_at | 0.811916 | 9.547657 | 4.452185 | 0.000209 | 0.010247 | 0.706838 | ARID5B |
| 212632_at | -0.66274 | 6.642274 | -4.89904 | 7.12E-05 | 0.005667 | 1.724479 | STX7 |
| 212665_at | 0.842071 | 8.463224 | 4.437961 | 0.000216 | 0.010463 | 0.674364 | TIPARP |
| 212702_s_at | -0.80762 | 7.559411 | -7.42706 | 2.27E-07 | 0.000309 | 7.112125 | BICD2 |
| 212764_at | 0.814212 | 7.529692 | 3.545191 | 0.001861 | 0.038584 | -1.34895 | ZEB1 |
| 212796_s_at | -0.61641 | 8.307848 | -6.30175 | 2.67E-06 | 0.001123 | 4.816334 | TBC1D2B |
| 212823_s_at | -0.63704 | 7.465682 | -4.31566 | 0.000291 | 0.012686 | 0.395132 | PLEKHG3 |
| 212977_at | -0.88629 | 5.189378 | -4.98058 | 5.86E-05 | 0.005137 | 1.909203 | ACKR3 |
| 212989_at | 0.841749 | 5.261581 | 4.246152 | 0.000344 | 0.014018 | 0.236497 | SGMS1 |
| 212993_at | -0.82011 | 6.575021 | -3.81507 | 0.000975 | 0.025522 | -0.74324 | NACC2 |
| 213097_s_at | 0.712027 | 8.183604 | 3.760868 | 0.001111 | 0.027644 | -0.86554 | DNAJC2 |
| 213134_x_at | 0.597262 | 7.849479 | 3.485323 | 0.002147 | 0.041684 | -1.48205 | BTG3 |
| 213239_at | 0.956072 | 6.334696 | 4.125075 | 0.000461 | 0.016603 | -0.03958 | PIBF1 |
| 213474_at | -0.63244 | 7.618737 | -5.2537 | 3.05E-05 | 0.003699 | 2.52465 | KCTD7//RABGEF1 |
| 213733_at | -0.74702 | 9.646209 | -4.24538 | 0.000345 | 0.014018 | 0.234737 | MYO1F |
| 213848_at | -0.73134 | 7.415519 | -6.1268 | 3.98E-06 | 0.001271 | 4.443053 | DUSP7 |
| 213849_s_at | -1.06705 | 6.066855 | -4.29103 | 0.000309 | 0.013044 | 0.338924 | PPP2R2B |
| 213878_at | 0.68893 | 8.067692 | 3.488687 | 0.00213 | 0.041574 | -1.47458 | PYROXD1 |
| 213915_at | -1.18708 | 10.69726 | -4.4815 | 0.000195 | 0.009831 | 0.773768 | NKG7 |
| 214252_s_at | 0.7955 | 7.109125 | 4.200038 | 0.000385 | 0.014966 | 0.131299 | CLN5 |
| 214450_at | -0.7718 | 10.31927 | -3.61405 | 0.001579 | 0.034959 | -1.19525 | CTSW |
| 214453_s_at | 1.731757 | 7.901186 | 4.212709 | 0.000373 | 0.014609 | 0.1602 | IFI44 |
| 214469_at | -1.00415 | 4.842916 | -4.62829 | 0.000137 | 0.008163 | 1.108652 | H2AC8 |
| 214567_s_at | -0.83309 | 6.579861 | -3.4172 | 0.002524 | 0.045669 | -1.63281 | XCL2//XCL1 |
| 214574_x_at | 0.620774 | 7.116118 | 4.145953 | 0.000439 | 0.016151 | 0.00799 | LST1 |
| 214617_at | -1.31657 | 11.40207 | -4.83905 | 8.22E-05 | 0.0061 | 1.58831 | PRF1 |
| 214623_at | -0.60175 | 6.819164 | -4.92961 | 6.62E-05 | 0.005381 | 1.793771 | FBXW4P1 |
| 214683_s_at | 0.932846 | 8.614987 | 5.083938 | 4.57E-05 | 0.004596 | 2.14277 | CLK1 |
| 214850_at | -0.92715 | 7.119561 | -4.38173 | 0.000248 | 0.01131 | 0.54598 | GUSBP14 |
| 214995_s_at | -0.92667 | 8.071295 | -4.88744 | 7.32E-05 | 0.005806 | 1.698166 | APOBEC3F//APOBEC3G |
| 215239_x_at | 0.690229 | 5.096606 | 4.789025 | 9.28E-05 | 0.006429 | 1.474631 | ZNF273 |
| 215599_at | -0.75917 | 7.21458 | -3.96153 | 0.000685 | 0.020712 | -0.41156 | GUSBP14//GUSBP3//GUSBP17 |
| 215779_s_at | -1.16274 | 4.989669 | -5.3522 | 2.42E-05 | 0.003315 | 2.745113 | H2BC8//H2BC11 |
| 215806_x_at | -1.09046 | 10.56214 | -4.8203 | 8.60E-05 | 0.006216 | 1.545725 | TRGC2//TRGV9//TARP |
| 215894_at | -1.68094 | 6.206438 | -9.19061 | 6.65E-09 | 0.000145 | 10.31906 | PTGDR |
| 216305_s_at | 0.637836 | 6.936849 | 3.627213 | 0.00153 | 0.034258 | -1.16578 | GCFC2 |
| 216834_at | 1.593745 | 7.751821 | 4.823608 | 8.54E-05 | 0.006211 | 1.553234 | RGS1 |
| 216920_s_at | -1.04929 | 10.90832 | -4.48666 | 0.000192 | 0.009767 | 0.785545 | TARP//TRGC2//TRGV9 |
| 217381_s_at | -0.71813 | 6.987243 | -4.067 | 0.000531 | 0.017618 | -0.17183 | TARP |
| 217627_at | -0.67461 | 6.423882 | -3.5264 | 0.001947 | 0.039451 | -1.39078 | ZNF573 |
| 217730_at | -0.63908 | 8.828779 | -5.92914 | 6.27E-06 | 0.001687 | 4.016503 | TMBIM1//MIR6513 |
| 217992_s_at | -1.01741 | 9.451471 | -3.96017 | 0.000687 | 0.020721 | -0.41465 | EFHD2 |
| 218100_s_at | 0.774621 | 6.792946 | 4.044943 | 0.00056 | 0.018087 | -0.22201 | IFT57 |
| 218214_at | -0.63836 | 7.195326 | -4.87477 | 7.55E-05 | 0.005842 | 1.669405 | ATG101 |
| 218272_at | -1.21664 | 7.926354 | -5.22914 | 3.24E-05 | 0.003774 | 2.469546 | TTC38 |
| 218309_at | -0.65563 | 6.376592 | -4.72546 | 0.000108 | 0.007097 | 1.330006 | CAMK2N1 |
| 218311_at | 0.632616 | 4.65835 | 3.607094 | 0.001606 | 0.03533 | -1.21079 | MAP4K3 |
| 218319_at | 1.215593 | 7.735225 | 5.680538 | 1.12E-05 | 0.002276 | 3.473172 | PELI1 |
| 218361_at | -0.67704 | 7.192774 | -5.12014 | 4.20E-05 | 0.004398 | 2.224402 | GOLPH3L |
| 218474_s_at | -0.61577 | 7.208394 | -4.65652 | 0.000128 | 0.007831 | 1.172983 | KCTD5 |
| 218522_s_at | -0.71132 | 6.961037 | -7.15212 | 4.08E-07 | 0.00045 | 6.568743 | MAP1S |
| 218638_s_at | -1.30025 | 8.200991 | -3.98051 | 0.000654 | 0.019979 | -0.36846 | SPON2//LOC100130872 |
| 218919_at | 0.691698 | 7.585839 | 3.695566 | 0.001299 | 0.031051 | -1.01248 | ZFAND1 |
| 218924_s_at | 0.759623 | 6.833999 | 5.102659 | 4.37E-05 | 0.004526 | 2.184993 | CTBS |
| 218927_s_at | -0.73052 | 8.677286 | -3.57202 | 0.001746 | 0.037336 | -1.28913 | CHST12 |
| 218929_at | 0.591102 | 7.801998 | 4.015808 | 0.000601 | 0.018954 | -0.28827 | CDKN2AIP |
| 219035_s_at | -0.65486 | 8.174138 | -5.63246 | 1.25E-05 | 0.002455 | 3.367273 | RNF34 |
| 219123_at | -0.67317 | 6.351671 | -4.21371 | 0.000372 | 0.0146 | 0.162479 | ZNF232 |
| 219177_at | 0.667776 | 5.955695 | 4.581706 | 0.000153 | 0.008566 | 1.00242 | BRIX1 |
| 219183_s_at | -0.62183 | 9.155142 | -7.67745 | 1.34E-07 | 0.000266 | 7.5968 | CYTH4 |
| 219243_at | -0.64927 | 9.918112 | -3.46555 | 0.00225 | 0.042889 | -1.52589 | GIMAP4 |
| 219248_at | 0.659848 | 7.044968 | 4.293361 | 0.000307 | 0.012999 | 0.344236 | THUMPD2 |
| 219304_s_at | -0.72027 | 5.717822 | -3.34998 | 0.002959 | 0.049861 | -1.78081 | PDGFD |
| 219405_at | -0.62751 | 7.371565 | -6.29579 | 2.71E-06 | 0.001123 | 4.803682 | TRIM68 |
| 219426_at | -0.58851 | 6.88382 | -3.61636 | 0.00157 | 0.034941 | -1.19007 | AGO3 |
| 219521_at | -0.83881 | 6.486253 | -3.79081 | 0.001033 | 0.026542 | -0.79802 | B3GAT1 |
| 219529_at | -1.12022 | 5.375335 | -5.26048 | 3.00E-05 | 0.003688 | 2.539859 | CLIC3 |
| 219551_at | 0.900112 | 5.896588 | 5.406759 | 2.12E-05 | 0.003079 | 2.866849 | EAF2 |
| 219566_at | -1.08392 | 7.464136 | -6.57345 | 1.45E-06 | 0.000806 | 5.387699 | PLEKHF1 |
| 219913_s_at | -0.7196 | 4.870785 | -4.11502 | 0.000473 | 0.016692 | -0.06248 | CRNKL1 |
| 219957_at | -0.5868 | 7.455455 | -3.72575 | 0.001208 | 0.029453 | -0.94462 | RUFY2 |
| 220059_at | 0.800434 | 4.97893 | 3.64857 | 0.001454 | 0.033128 | -1.11794 | STAP1 |
| 220307_at | -1.01672 | 7.369179 | -5.27676 | 2.89E-05 | 0.003664 | 2.576333 | CD244 |
| 220684_at | -1.31935 | 7.861868 | -4.78854 | 9.29E-05 | 0.006429 | 1.473518 | TBX21 |
| 220739_s_at | -0.63556 | 8.400066 | -5.09126 | 4.49E-05 | 0.00458 | 2.159282 | CNNM3 |
| 220918_at | -0.99691 | 7.571828 | -7.01872 | 5.45E-07 | 0.00054 | 6.300925 | RUNX1-IT1 |
| 220934_s_at | -0.73823 | 8.014613 | -4.98585 | 5.78E-05 | 0.005116 | 1.921139 | TMEM223 |
| 220940_at | -0.63958 | 8.343701 | -3.36596 | 0.002849 | 0.048805 | -1.74571 | ANKRD36B |
| 221081_s_at | -0.64798 | 10.39986 | -7.20476 | 3.64E-07 | 0.00045 | 6.673678 | DENND2D |
| 221191_at | -0.72767 | 7.191307 | -4.58695 | 0.000151 | 0.008564 | 1.014372 | STAG3L1//STAG3L3 |
| 221565_s_at | -1.19717 | 7.690006 | -7.57553 | 1.66E-07 | 0.0003 | 7.400697 | CALHM2 |
| 221763_at | 0.950679 | 7.735801 | 4.117499 | 0.00047 | 0.016685 | -0.05684 | JMJD1C |
| 221830_at | -0.89493 | 8.466275 | -4.61842 | 0.00014 | 0.008225 | 1.086153 | RAP2A |
| 221840_at | -0.63865 | 7.950488 | -4.0032 | 0.000619 | 0.019186 | -0.31693 | PTPRE |
| 221883_at | -0.64186 | 6.628331 | -4.9853 | 5.79E-05 | 0.005116 | 1.919897 | PKNOX1 |
| 222067_x_at | -1.02496 | 7.171698 | -4.25997 | 0.000333 | 0.01372 | 0.268023 | H2BC5 |
| 222217_s_at | -0.85618 | 6.902428 | -5.00335 | 5.55E-05 | 0.005069 | 1.960736 | SLC27A3 |
| 222243_s_at | -1.11525 | 7.211181 | -6.78292 | 9.13E-07 | 0.000622 | 5.820961 | TOB2 |
| 222420_s_at | -0.67872 | 7.740928 | -4.07433 | 0.000522 | 0.017441 | -0.15515 | UBE2H |
| 222538_s_at | -0.69891 | 6.392282 | -3.66517 | 0.001397 | 0.032241 | -1.08072 | APPL1 |
| 222607_s_at | 0.715606 | 6.366038 | 4.190979 | 0.000393 | 0.014968 | 0.11064 | DIS3 |
| 222617_s_at | 0.734217 | 7.570828 | 3.529991 | 0.00193 | 0.039279 | -1.38279 | FAM204A |
| 222669_s_at | 0.905368 | 9.355586 | 4.25302 | 0.000338 | 0.01389 | 0.25217 | SBDS//SBDSP1 |
| 222698_s_at | 0.626094 | 4.785591 | 3.434429 | 0.002423 | 0.044581 | -1.59476 | IMPACT |
| 222699_s_at | 0.592729 | 7.730339 | 5.328792 | 2.55E-05 | 0.003377 | 2.692803 | PLEKHF2 |
| 222792_s_at | 0.82113 | 8.257287 | 4.794924 | 9.15E-05 | 0.006393 | 1.488042 | CCDC59 |
| 222838_at | -1.21941 | 7.649757 | -4.22823 | 0.000359 | 0.014405 | 0.195616 | SLAMF7 |
| 222985_at | -0.84711 | 8.105329 | -6.84451 | 7.97E-07 | 0.000599 | 5.947137 | YWHAG |
| 223060_at | -0.89948 | 7.944956 | -5.39985 | 2.16E-05 | 0.003079 | 2.85144 | C14orf119 |
| 223081_at | -0.98673 | 7.80365 | -7.43479 | 2.23E-07 | 0.000309 | 7.127235 | PHF23 |
| 223096_at | 0.708056 | 8.557264 | 3.777056 | 0.001068 | 0.026934 | -0.82905 | NOP58 |
| 223125_s_at | -1.4432 | 6.751289 | -4.4521 | 0.000209 | 0.010247 | 0.706653 | C1orf21 |
| 223132_s_at | -0.69419 | 8.912116 | -5.0301 | 5.20E-05 | 0.004883 | 2.021206 | TRIM8 |
| 223154_at | 0.611602 | 7.679295 | 3.638082 | 0.001491 | 0.033667 | -1.14144 | MRPL1 |
| 223168_at | -0.90275 | 6.291838 | -3.61095 | 0.001591 | 0.035183 | -1.20217 | RHOU |
| 223195_s_at | -0.65313 | 7.53928 | -5.32977 | 2.55E-05 | 0.003377 | 2.694986 | SESN2 |
| 223209_s_at | -0.63677 | 7.773551 | -4.71168 | 0.000112 | 0.007167 | 1.298633 | SELENOS |
| 223218_s_at | 0.918681 | 9.887865 | 4.145044 | 0.00044 | 0.016151 | 0.005918 | NFKBIZ |
| 223231_at | 0.855407 | 7.648276 | 3.86237 | 0.00087 | 0.0241 | -0.63631 | TATDN1 |
| 223267_at | 0.820404 | 7.026866 | 4.594402 | 0.000148 | 0.008487 | 1.031377 | TRMT10C |
| 223276_at | -0.71545 | 5.966416 | -4.4686 | 0.000201 | 0.009981 | 0.744313 | SMIM3 |
| 223297_at | -0.6498 | 7.08218 | -5.12021 | 4.19E-05 | 0.004398 | 2.224544 | AMMECR1L |
| 223298_s_at | 0.704559 | 8.748543 | 3.779311 | 0.001062 | 0.026933 | -0.82396 | NT5C3A |
| 223344_s_at | 0.862766 | 6.144434 | 4.086183 | 0.000507 | 0.017194 | -0.12816 | MS4A7 |
| 223377_x_at | -1.10179 | 9.6534 | -4.08789 | 0.000505 | 0.017194 | -0.12427 | CISH |
| 223401_at | 1.007781 | 8.371503 | 5.761403 | 9.25E-06 | 0.002034 | 3.650701 | ADPRM |
| 223424_s_at | -0.81592 | 6.584758 | -4.29438 | 0.000306 | 0.012999 | 0.346557 | ZSCAN21 |
| 223501_at | 1.267572 | 6.628521 | 4.397459 | 0.000239 | 0.011085 | 0.581891 | TNFSF13B |
| 223526_at | 0.60188 | 7.727055 | 4.180839 | 0.000403 | 0.015233 | 0.087516 | C18orf21 |
| 223566_s_at | -0.7965 | 6.730532 | -3.35247 | 0.002941 | 0.049861 | -1.77535 | BCOR |
| 223583_at | -0.6621 | 7.023345 | -4.53068 | 0.000173 | 0.009171 | 0.886001 | TNFAIP8L2 |
| 223615_at | -0.69385 | 7.549057 | -5.45123 | 1.91E-05 | 0.002917 | 2.965862 | ABI3 |
| 223714_at | 0.934628 | 5.415107 | 5.590097 | 1.38E-05 | 0.002572 | 3.273752 | ZNF256 |
| 223836_at | -1.60155 | 9.480659 | -3.5999 | 0.001633 | 0.035676 | -1.22688 | FGFBP2 |
| 223887_at | -0.66397 | 6.182346 | -5.07753 | 4.64E-05 | 0.004646 | 2.128312 | GPR132 |
| 224285_at | -0.62617 | 5.069418 | -4.18932 | 0.000395 | 0.015002 | 0.10686 | GPR174 |
| 224367_at | 0.827576 | 8.772813 | 4.168041 | 0.000416 | 0.015631 | 0.058338 | BEX2 |
| 224504_s_at | -0.79047 | 6.965184 | -5.73007 | 9.95E-06 | 0.002138 | 3.582001 | BUD13 |
| 224610_at | 0.822487 | 10.66038 | 4.593223 | 0.000149 | 0.008488 | 1.028687 | SNHG1//SNORD29//SNORD31//SNORD30//SNORD28//SNORD27//SNORD26//SNORD25//SNORD22 |
| 224708_at | -0.74965 | 7.699927 | -5.86967 | 7.20E-06 | 0.001813 | 3.887193 | KIAA2013 |
| 224748_at | -0.81855 | 8.299403 | -4.38214 | 0.000248 | 0.01131 | 0.546912 | DCAF7 |
| 224789_at | -0.65335 | 7.082672 | -4.87438 | 7.56E-05 | 0.005842 | 1.668523 | DCAF12 |
| 224793_s_at | -0.60408 | 8.649482 | -4.25255 | 0.000339 | 0.01389 | 0.251098 | TGFBR1 |
| 224797_at | 1.059421 | 7.508821 | 4.080653 | 0.000514 | 0.017248 | -0.14075 | ARRDC3 |
| 224826_at | 0.607787 | 8.973875 | 4.062275 | 0.000537 | 0.017686 | -0.18258 | GPCPD1 |
| 224881_at | -0.69704 | 6.043368 | -4.95981 | 6.16E-05 | 0.005243 | 1.862202 | VKORC1L1 |
| 224927_at | -0.74299 | 10.77279 | -5.86536 | 7.27E-06 | 0.001813 | 3.877814 | PPP1R18 |
| 224962_at | -0.82412 | 7.730946 | -7.98251 | 7.14E-08 | 0.00026 | 8.174131 | TMEM250 |
| 225005_at | -0.66302 | 7.156565 | -5.92424 | 6.34E-06 | 0.001687 | 4.005853 | PHF13 |
| 225043_at | -0.76058 | 7.933433 | -5.77942 | 8.87E-06 | 0.002025 | 3.690153 | SLC15A4 |
| 225086_at | -0.67405 | 6.336484 | -4.08112 | 0.000513 | 0.017248 | -0.13968 | FAM98B |
| 225099_at | -0.86531 | 6.24599 | -5.01455 | 5.40E-05 | 0.004989 | 1.986046 | FBXO45 |
| 225105_at | -0.63915 | 8.405331 | -3.65129 | 0.001444 | 0.033016 | -1.11184 | C12orf75 |
| 225127_at | -0.68588 | 8.506459 | -5.34061 | 2.48E-05 | 0.003344 | 2.71923 | TMEM181 |
| 225219_at | -0.77291 | 5.979515 | -3.53284 | 0.001917 | 0.039151 | -1.37645 | SMAD5 |
| 225253_s_at | -0.60824 | 7.152911 | -4.52182 | 0.000177 | 0.009307 | 0.865778 | METTL2A//METTL2B |
| 225496_s_at | -0.67378 | 8.026476 | -3.42477 | 0.002479 | 0.045194 | -1.6161 | SYTL2 |
| 225525_at | -0.96617 | 7.324282 | -4.71254 | 0.000112 | 0.007167 | 1.300593 | KIAA1671 |
| 225530_at | -0.87919 | 9.648786 | -7.71395 | 1.24E-07 | 0.000266 | 7.666645 | MOB3A |
| 225615_at | -0.59279 | 7.973179 | -6.2511 | 3.00E-06 | 0.001168 | 4.708703 | IFFO2 |
| 225644_at | -0.76593 | 6.756828 | -4.11765 | 0.00047 | 0.016685 | -0.05649 | CCDC117 |
| 225673_at | -0.86483 | 8.991892 | -6.18032 | 3.52E-06 | 0.001221 | 4.557687 | MYADM |
| 225679_at | -0.72816 | 6.300078 | -4.53033 | 0.000173 | 0.009171 | 0.885198 | NAA30 |
| 225798_at | -0.61288 | 7.651134 | -3.70055 | 0.001284 | 0.03086 | -1.00129 | JAZF1 |
| 225848_at | -0.60496 | 7.639969 | -7.85784 | 9.22E-08 | 0.00026 | 7.93995 | ZNF746 |
| 225951_s_at | 0.615843 | 10.62523 | 4.120686 | 0.000466 | 0.016685 | -0.04958 | CHASERR |
| 225974_at | -0.91246 | 6.657598 | -5.15773 | 3.84E-05 | 0.004246 | 2.309047 | TMEM64 |
| 226032_at | -0.61099 | 9.241125 | -5.24715 | 3.10E-05 | 0.003736 | 2.509966 | CASP2 |
| 226092_at | 0.608173 | 6.299445 | 3.755141 | 0.001126 | 0.027868 | -0.87845 | MPP5 |
| 226337_at | 0.624707 | 5.58116 | 3.4161 | 0.00253 | 0.04575 | -1.63525 | GORAB |
| 226348_at | -1.03777 | 7.877719 | -5.99413 | 5.40E-06 | 0.001502 | 4.157285 | FUT11 |
| 226423_at | -0.85286 | 7.689636 | -5.40132 | 2.15E-05 | 0.003079 | 2.85473 | PAQR8 |
| 226515_at | -0.58829 | 7.471276 | -3.65072 | 0.001446 | 0.033027 | -1.11312 | CCDC127 |
| 226548_at | -0.66963 | 8.495042 | -4.58154 | 0.000153 | 0.008566 | 1.002037 | SBK1 |
| 226554_at | -0.63736 | 9.460342 | -6.32039 | 2.56E-06 | 0.001118 | 4.855875 | ZBTB7A |
| 226608_at | 0.945725 | 5.729887 | 4.121698 | 0.000465 | 0.016685 | -0.04728 | C16orf87 |
| 226633_at | -0.59952 | 9.021966 | -5.10219 | 4.38E-05 | 0.004526 | 2.183945 | RAB8B |
| 226673_at | -0.70402 | 8.471092 | -6.40262 | 2.13E-06 | 0.001032 | 5.029672 | SH2D3C |
| 226743_at | -0.59056 | 5.657914 | -4.10234 | 0.000487 | 0.016949 | -0.09138 | SLFN11 |
| 226838_at | 0.589308 | 8.303906 | 4.266485 | 0.000328 | 0.013557 | 0.282895 | TTC32 |
| 226977_at | -0.6475 | 6.716267 | -4.22143 | 0.000365 | 0.01446 | 0.180104 | IGIP |
| 226991_at | -0.68171 | 9.567629 | -4.78162 | 9.44E-05 | 0.006475 | 1.457795 | NFATC2 |
| 227013_at | -0.91531 | 5.793778 | -3.50961 | 0.002026 | 0.04046 | -1.42811 | LATS2 |
| 227030_at | -0.66538 | 9.874012 | -5.16328 | 3.79E-05 | 0.004212 | 2.321529 | IKZF3 |
| 227052_at | -0.69258 | 7.520189 | -5.55145 | 1.51E-05 | 0.002658 | 3.188258 | SMIM14 |
| 227066_at | -0.65608 | 6.750903 | -5.50021 | 1.70E-05 | 0.002774 | 3.074685 | MOB3C |
| 227309_at | 0.684305 | 6.201916 | 3.965787 | 0.000678 | 0.020529 | -0.4019 | YOD1 |
| 227348_at | -0.60021 | 5.33371 | -3.92074 | 0.000756 | 0.022059 | -0.50411 | PARS2 |
| 227384_s_at | -0.69766 | 10.4839 | -4.83268 | 8.35E-05 | 0.006152 | 1.573845 | LINC00869 |
| 227410_at | -0.65076 | 6.667778 | -3.782 | 0.001056 | 0.026871 | -0.8179 | FAM43A |
| 227446_s_at | -0.59016 | 6.484073 | -4.34632 | 0.00027 | 0.012069 | 0.465138 | DHRS4-AS1 |
| 227521_at | 0.80918 | 8.175236 | 4.300682 | 0.000302 | 0.012963 | 0.360948 | FBXO33 |
| 227577_at | 0.838017 | 6.843777 | 3.519616 | 0.001978 | 0.039835 | -1.40587 | EXOC8 |
| 227639_at | 0.606842 | 6.86915 | 3.74091 | 0.001165 | 0.02861 | -0.9105 | PIGK |
| 227793_at | 0.594608 | 7.277749 | 4.480337 | 0.000195 | 0.009836 | 0.771103 | MIRLET7D |
| 228062_at | 0.794806 | 6.285451 | 3.665309 | 0.001397 | 0.032241 | -1.08041 | NAP1L5 |
| 228087_at | -0.73437 | 5.859296 | -5.35564 | 2.40E-05 | 0.003315 | 2.752795 | CCDC126 |
| 228098_s_at | 0.65243 | 10.45043 | 4.550679 | 0.000165 | 0.008895 | 0.931637 | MYLIP |
| 228113_at | -0.66957 | 8.694712 | -5.99078 | 5.44E-06 | 0.001502 | 4.150055 | RAB37 |
| 228189_at | -0.84626 | 6.298439 | -5.90055 | 6.70E-06 | 0.001739 | 3.954391 | BAG4 |
| 228258_at | -0.61838 | 10.89428 | -5.76835 | 9.10E-06 | 0.002025 | 3.665909 | TBC1D10C |
| 228259_s_at | 0.680729 | 6.154554 | 5.085144 | 4.56E-05 | 0.004596 | 2.14549 | EPB41L4A//EPB41L4A-AS1 |
| 228372_at | -0.76078 | 7.324928 | -3.37938 | 0.00276 | 0.047974 | -1.71619 | TMEM273 |
| 228381_at | 1.150449 | 8.813695 | 4.529556 | 0.000173 | 0.009171 | 0.883439 | ATF7IP2 |
| 228410_at | -0.65833 | 7.967316 | -3.89216 | 0.00081 | 0.023138 | -0.56886 | GAB3 |
| 228528_at | 0.5981 | 6.393076 | 3.39978 | 0.00263 | 0.046681 | -1.67125 | MIR29B2CHG//MIR29B2//MIR29C |
| 228543_at | 0.869003 | 6.534203 | 3.56912 | 0.001758 | 0.037512 | -1.29561 | PET117 |
| 228562_at | 0.717496 | 5.654755 | 3.351141 | 0.002951 | 0.049861 | -1.77827 | ZBTB10 |
| 228617_at | 1.333635 | 8.927665 | 3.390266 | 0.00269 | 0.047353 | -1.69221 | XAF1 |
| 228654_at | -0.76104 | 3.720621 | -4.1801 | 0.000404 | 0.015234 | 0.085834 | SPIN4 |
| 228723_at | -0.73063 | 5.881541 | -5.26862 | 2.95E-05 | 0.003672 | 2.558097 | NPTN-IT1 |
| 228745_at | 0.713704 | 7.593852 | 3.893767 | 0.000806 | 0.023103 | -0.56523 | SGTB |
| 228788_at | -0.9989 | 6.598775 | -4.93324 | 6.56E-05 | 0.005378 | 1.802005 | YPEL1 |
| 228920_at | -0.63332 | 6.095519 | -3.35283 | 0.002939 | 0.049861 | -1.77456 | ZNF260 |
| 228927_at | -0.67248 | 6.357054 | -5.05617 | 4.89E-05 | 0.004757 | 2.080102 | ZNF397 |
| 229055_at | -0.87098 | 7.213219 | -6.50544 | 1.69E-06 | 0.000878 | 5.245654 | GPR68 |
| 229121_at | -0.72568 | 6.228011 | -3.99582 | 0.00063 | 0.019362 | -0.33369 | CMKLR1 |
| 229215_at | -1.11971 | 6.3409 | -4.09969 | 0.00049 | 0.017031 | -0.09741 | ASCL2 |
| 229722_at | 0.754855 | 6.348613 | 5.313295 | 2.65E-05 | 0.003438 | 2.658142 | LINC00653 |
| 229971_at | -1.20572 | 6.260286 | -5.26908 | 2.94E-05 | 0.003672 | 2.559127 | ADGRG5 |
| 230252_at | -1.0062 | 5.217397 | -4.50491 | 0.000184 | 0.009533 | 0.827194 | LPAR5 |
| 230256_at | -0.64698 | 5.517592 | -4.64894 | 0.00013 | 0.00794 | 1.155722 | RUSC1-AS1 |
| 230464_at | -1.71633 | 8.451303 | -5.65699 | 1.18E-05 | 0.002361 | 3.42134 | S1PR5 |
| 230563_at | -1.35874 | 6.216653 | -6.22049 | 3.22E-06 | 0.00118 | 4.643463 | RASGEF1A |
| 230756_at | -1.06411 | 7.595477 | -4.19514 | 0.000389 | 0.014966 | 0.120127 | ZNF683 |
| 230836_at | -0.61356 | 5.556855 | -3.82877 | 0.000943 | 0.025329 | -0.7123 | ST8SIA4 |
| 230860_at | -0.61621 | 4.971707 | -3.88941 | 0.000815 | 0.023204 | -0.5751 | CEP19 |
| 230925_at | -0.64304 | 10.61845 | -5.5659 | 1.46E-05 | 0.002619 | 3.220248 | APBB1IP |
| 231252_at | -0.94633 | 5.942263 | -4.74905 | 0.000102 | 0.006863 | 1.383712 | KANSL1L |
| 231638_at | -0.78901 | 6.102805 | -5.19814 | 3.48E-05 | 0.00402 | 2.399925 | PRSS30P |
| 231776_at | -1.22923 | 9.033219 | -7.53905 | 1.79E-07 | 0.0003 | 7.330114 | EOMES |
| 231911_at | 0.71802 | 4.985771 | 3.817641 | 0.000969 | 0.025514 | -0.73744 | ERMN |
| 231989_s_at | -0.81275 | 7.630261 | -3.70143 | 0.001281 | 0.030829 | -0.99931 | NPIPB3//SLC7A5P1//NPIPB4//NPIPB13//SMG1P1//NPIPB5//SMG1P3//NPIPA5 |
| 232028_at | -0.98669 | 5.910409 | -6.04423 | 4.81E-06 | 0.001437 | 4.265467 | ZNF678 |
| 232051_at | -0.69318 | 6.132117 | -6.88916 | 7.23E-07 | 0.000584 | 6.038233 | CCDC102A |
| 232284_at | 0.700888 | 6.216657 | 4.47207 | 0.000199 | 0.009966 | 0.752232 | PSMD6//PSMD6-AS2 |
| 232346_at | -0.73491 | 8.094634 | -4.11848 | 0.000469 | 0.016685 | -0.05461 | LINC02591 |
| 232686_at | -1.51937 | 6.691795 | -5.53924 | 1.56E-05 | 0.002703 | 3.16122 | SIGLEC17P |
| 233955_x_at | -0.63971 | 7.858817 | -3.89054 | 0.000813 | 0.023198 | -0.57254 | CXXC5 |
| 234594_at | -0.61885 | 5.125184 | -4.87708 | 7.51E-05 | 0.005842 | 1.674651 | ITPK1-AS1 |
| 234983_at | -0.58934 | 7.368873 | -3.799 | 0.001013 | 0.026248 | -0.77953 | SPRING1 |
| 235046_at | 0.780705 | 7.475106 | 4.333666 | 0.000278 | 0.012293 | 0.436245 | INPP4B |
| 235060_at | -0.78021 | 8.643018 | -3.64016 | 0.001483 | 0.033557 | -1.1368 | LOC100190986 |
| 235085_at | -0.84806 | 8.982815 | -4.964 | 6.09E-05 | 0.005211 | 1.871683 | PRAG1 |
| 235170_at | 0.752791 | 5.961505 | 3.844831 | 0.000907 | 0.024702 | -0.67599 | ZNF92 |
| 235192_at | -0.5959 | 7.153335 | -4.63166 | 0.000136 | 0.008119 | 1.116327 | TP53RK |
| 235593_at | -1.01197 | 5.165207 | -3.96825 | 0.000674 | 0.020436 | -0.39632 | ZEB2 |
| 235651_at | -0.9037 | 7.23202 | -5.26114 | 3.00E-05 | 0.003688 | 2.541333 | TTC22 |
| 235837_at | -0.99728 | 6.379187 | -5.23509 | 3.19E-05 | 0.003774 | 2.482894 | SNIP1 |
| 236295_s_at | -0.59483 | 9.92784 | -6.59075 | 1.40E-06 | 0.000802 | 5.423717 | NLRC3 |
| 236341_at | 0.915003 | 7.084182 | 3.871489 | 0.000851 | 0.023869 | -0.61568 | CTLA4 |
| 236526_x_at | 0.942235 | 4.761051 | 4.103382 | 0.000486 | 0.016934 | -0.089 | TRAPPC13 |
| 236833_at | -0.97408 | 7.14879 | -5.47664 | 1.80E-05 | 0.002863 | 3.022341 | TTC16 |
| 238035_at | 0.657294 | 8.564273 | 5.149892 | 3.91E-05 | 0.004282 | 2.291405 | SP3 |
| 238360_s_at | -0.98897 | 5.932731 | -6.44043 | 1.96E-06 | 0.000969 | 5.109262 | LINC00672 |
| 238673_at | 0.70385 | 4.735999 | 3.453445 | 0.002316 | 0.04368 | -1.55269 | SAMD12 |
| 238695_s_at | -0.78717 | 5.497939 | -4.23886 | 0.00035 | 0.014145 | 0.219865 | RAB39B |
| 238722_x_at | -0.70957 | 5.406344 | -4.22469 | 0.000363 | 0.014455 | 0.187532 | NAPEPLD |
| 238788_at | -0.9506 | 5.289376 | -5.62679 | 1.27E-05 | 0.002464 | 3.354763 | PHBP19 |
| 239237_at | -0.86434 | 9.064784 | -6.99009 | 5.80E-07 | 0.00055 | 6.243089 | TRG-AS1 |
| 239294_at | -0.72212 | 8.045964 | -5.55646 | 1.49E-05 | 0.002648 | 3.199354 | PIK3CG |
| 242304_at | 0.626864 | 7.946761 | 3.550426 | 0.001838 | 0.03838 | -1.33729 | PYM1 |
| 243124_at | -0.86535 | 6.858086 | -5.26889 | 2.94E-05 | 0.003672 | 2.558698 | RRN3P2 |
| 244766_at | -1.27815 | 6.499165 | -6.04665 | 4.79E-06 | 0.001437 | 4.270683 | SMG1//SLC7A5P1//NPIPB4//SMG1P2//BOLA2//SMG1P5//NPIPB13//SMG1P1//NPIPB5//SMG1P3//NPIPA5//SMG1P7 |
| 31845_at | -0.6637 | 8.385253 | -4.67213 | 0.000123 | 0.007593 | 1.208551 | ELF4 |
| 32091_at | -0.71559 | 7.055533 | -4.29034 | 0.000309 | 0.013044 | 0.337337 | SLC25A44 |
| 35160_at | -0.64587 | 7.982717 | -3.67448 | 0.001366 | 0.031799 | -1.05982 | LDB1 |
| 47069_at | -0.62703 | 8.825227 | -5.9992 | 5.34E-06 | 0.001502 | 4.168259 | PRR5 |
| 53968_at | -0.73524 | 7.946248 | -4.93474 | 6.54E-05 | 0.005378 | 1.805406 | INTS5 |
| LncRNAs are highlighted in yellow | | | | | | | |
